# XIST and MUC1-C form an auto-regulatory pathway in driving cancer progression

**DOI:** 10.1038/s41419-024-06684-9

**Published:** 2024-05-13

**Authors:** Keyi Wang, Atrayee Bhattacharya, Naoki Haratake, Tatsuaki Daimon, Ayako Nakashoji, Hiroki Ozawa, Bo Peng, Wei Li, Donald Kufe

**Affiliations:** 1 Dana-Farber Cancer Institute, Harvard Medical School, Boston, MA USA; 2 Department of Urology, Shanghai Tenth People’s Hospital, Tongji University, Shanghai, China

**Keywords:** Biological sciences, Cancer genetics

## Abstract

The long non-coding RNA X-inactive specific transcript (lncRNA XIST) and *MUC1* gene are dysregulated in chronic inflammation and cancer; however, there is no known interaction of their functions. The present studies demonstrate that MUC1-C regulates XIST lncRNA levels by suppressing the RBM15/B, WTAP and METTL3/14 components of the m6A methylation complex that associate with XIST A repeats. MUC1-C also suppresses the YTHDF2-CNOT1 deadenylase complex that recognizes m6A sites and contributes to XIST decay with increases in XIST stability and expression. In support of an auto-regulatory pathway, we show that XIST regulates MUC1-C expression by promoting NF-κB-mediated activation of the *MUC1* gene. Of significance, MUC1-C and XIST regulate common genes associated with inflammation and stemness, including (i) miR-21 which is upregulated across pan-cancers, and (ii) TDP-43 which associates with the XIST E repeats. Our results further demonstrate that the MUC1-C/XIST pathway (i) is regulated by TDP-43, (ii) drives stemness-associated genes, and (iii) is necessary for self-renewal capacity. These findings indicate that the MUC1-C/XIST auto-regulatory axis is of importance in cancer progression.

## Introduction

The lncRNA XIST initiates X chromosome inactivation (XCI) in female placental mammals to balance dosage of X-linked gene expression between the sexes [1]. In executing phases of XCI, repeat arrays in the XIST RNA interact with diverse effectors that include the m6A methylation complex, epigenetic modifiers, and RNA splicing factors [2]. In addition to initiating and executing XCI, XIST plays a role in the inflammatory response of post-XCI somatic cells. XIST responds to acute inflammation by regulating NF-κB activity [3, 4]. XIST is also dysregulated in settings of chronic inflammation, including atherosclerosis [5] and neurodegenerative diseases [6]. Dysregulation of *XIST* expression has been recognized across numerous female and male cancers in association with both tumor progression and suppression [7–9]. Studies in breast cancer cells have implicated XIST overexpression with loss of the inactive X chromosome (Xi) or Barr body and increased transcription of the active X chromosome [10–12]. Other work has indicated that the XCI transcriptional program can be selectively accessed in male cancers [13]. Aberrant overexpression of XIST in cancer largely functions in promoting oncogenesis by regulating inflammation and diverse miRNAs that contribute to the CSC state and drug resistance [9, 14, 15]. In contrast to studies of XCI, less is known about the regulation of XIST, the effectors that bind to the XIST array repeats and downstream target genes in cancer cells. The potential involvement of XIST in contributing to intrinsic chronic inflammation in cancer progression also remains unclear.

The *MUC1* gene evolved in mammals for the protection of barrier tissues from loss of homeostasis [16–18]. *MUC1* encodes a transmembrane MUC1-C subunit that is activated by inflammation and contributes to barrier tissue stem cell functions associated with wound healing [17, 18]. MUC1-C induces lineage plasticity, the epithelial-mesenchymal transition (EMT), epigenetic reprogramming, and chromatin remodeling, which are of importance for repair and memory responses to inflammation [17–20]. These responses are in principle reversible with repair; however, persistent MUC1-C activation in settings of chronic inflammation can promote progression of the cancer stem cell (CSC) state [17, 18]. MUC1-C is activated by the inflammatory IKK→NF-κB pathway in cancer cells [21, 22]. In an auto-inductive pathway, MUC1-C binds directly to NF-κB and promotes activation of NF-κB target genes that include *MUC1* itself [21, 22]. In turn, MUC1-C interacts with other transcription factors (TFs), such as MYC and E2Fs, in activating the (i) Polycomb Repressive Complexes 1/2 (PRC1/2), and (ii) SWI/SNF embryonic stem cell esBAF and poly-bromo PBAF complexes [20, 23–26]. In this way, MUC1-C drives global changes in chromatin accessibility across the genomes of cancer cells [27]. Moreover, CSCs derived from diverse cancers are dependent on MUC1-C for chromatin remodeling, self-renewal and treatment resistance [18, 25–32]. Little is known about MUC1-C involvement in the regulation of long non-coding RNAs (lncRNAs) [33].

MUC1-C and XIST are dysregulated in chronic inflammation and cancer [9, 18]. There is no known association of MUC1-C and XIST in promoting cancer progression. The present results demonstrate that MUC1-C upregulates XIST expression in male and female cancer cells. Moreover, XIST activates the *MUC1* gene and thereby increases MUC1-C expression. In support of this MUC1-C/XIST auto-inductive pathway, we show that MUC1-C and XIST regulate common sets of genes associated with chronic inflammation and cancer progression.

## Results

### MUC1-C is necessary for XIST expression in human cancer cells

**Fig. 1:**
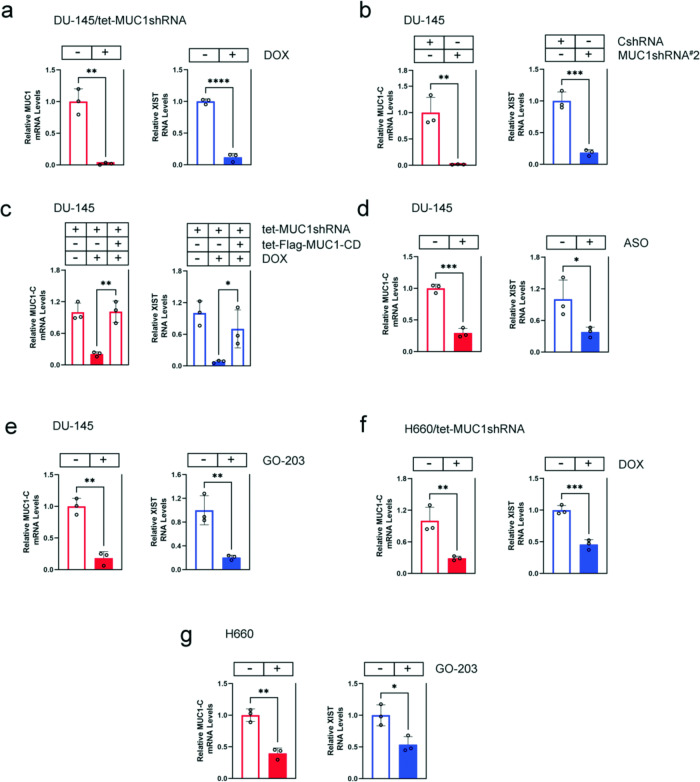
Targeting MUC1-C suppresses XIST expression. **a** DU-145/tet-MUC1shRNA cells treated with vehicle or DOX for 7 days were analyzed for MUC1-C mRNA and XIST RNA levels by qRT-PCR using primers listed in Supplementary Table S1. **b** DU-145/CshRNA and DU-145/MUC1shRNA#2 cells were analyzed for MUC1-C mRNA and XIST RNA levels. **c** DU-145 cells expressing the indicated vectors were treated with vehicle or DOX for 10 days and analyzed for MUC1-C mRNA and XIST RNA levels. **d** DU-145 cells transfected with 30 nM control/ASO or MUC1-C/ASO for 24 h were analyzed for MUC1-C mRNA and XIST RNA levels. **e** DU-145 cells treated with 5 μM GO-203 for 24 h were analyzed for MUC1-C mRNA and XIST RNA levels. **f** H660/tet-MUC1shRNA cells treated with vehicle or DOX for 7 days were analyzed for MUC1-C mRNA and XIST RNA levels. **g** H660 cells treated with 5 μM GO-203 for 24 h were analyzed for MUC1-C mRNA and XIST RNA levels. The results (mean ± SD of three determinations) are expressed as relative transcript levels compared to that obtained for control cells (assigned a value of 1).

XIST is upregulated in male and female cancers by unclear mechanisms [9]. To determine if MUC1-C contributes to XIST dysregulation, we studied DU-145 castration-resistant prostate cancer (CRPC) cells expressing a tet-CshRNA or tet-MUC1shRNA vector [28]. DOX treatment of DU-145/tet-MUC1shRNA, but not DU-145/tet-CshRNA, cells was associated with suppression of XIST RNA levels (Fig. 1a; Supplementary Fig. S1a). To exclude potential off-target effects, studies were performed on DU-145 cells expressing a second MUC1shRNA#2, which similarly demonstrated downregulation of XIST transcripts (Fig. 1b). As a control, rescue of MUC1-C silencing by DOX-inducible expression of a Flag-tagged MUC1-C cytoplasmic domain (tet-Flag-MUC1-CD) reversed XIST suppression (Fig. 1c). These results were extended by demonstrating that targeting MUC1-C with an anti-sense oligonucleotide (ASO) also decreases XIST levels (Fig. 1d). The MUC1-C cytoplasmic domain contains a CQC motif that is necessary for MUC1-C homodimerization and function [17]. Treatment of DU-145 cells with the GO-203 inhibitor, which blocks the CQC motif [17], suppressed XIST transcripts, confirming that MUC1-C increases XIST expression (Fig. 1e). These results were not limited to DU-145 cells in that targeting MUC1-C in male H660 neuroendocrine prostate cancer (NEPC) cells downregulated XIST levels (Fig. 1f, g). In addition, we studied female MDA-MB-436 and MDA-MB-468 triple-negative breast cancer (TNBC) cells, which express XIST (Supplementary Fig. S1b), and found that silencing MUC1-C decreases XIST expression (Supplementary Fig. S1c, d). These findings indicated that MUC1-C regulates XIST in cancers derived from both sexes.

### MUC1-C stabilizes XIST by suppressing XIST m6A methylation

**Fig. 2:**
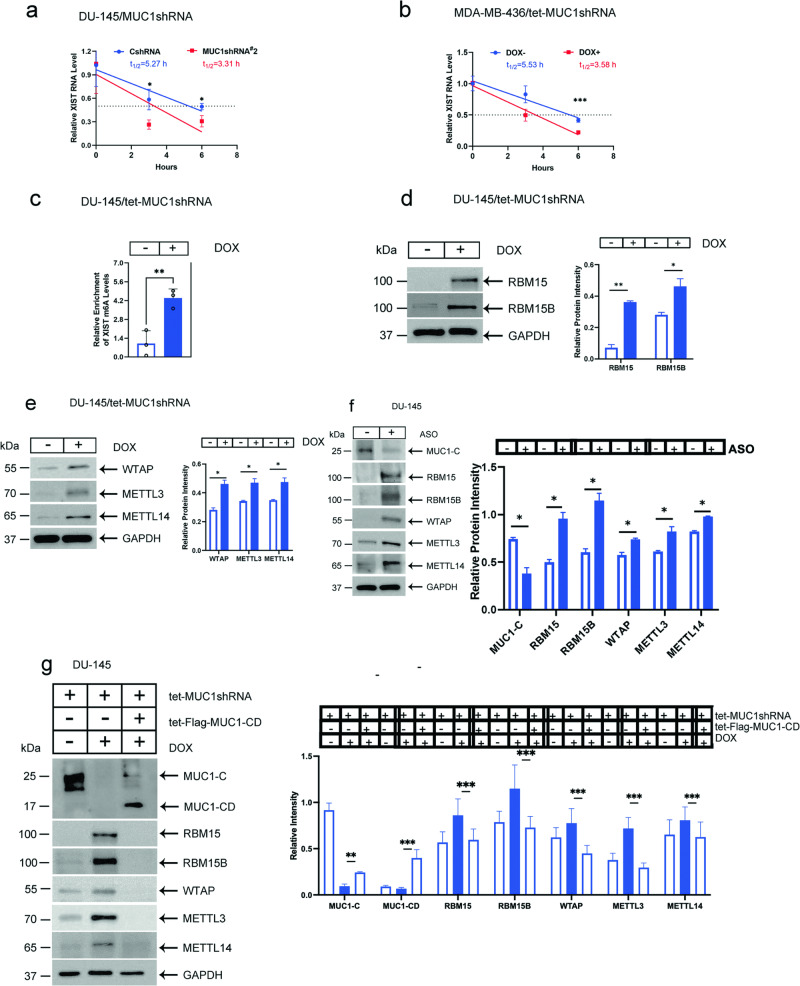
MUC1-C suppresses XIST m6A methylation and expression of the m6A methylation complex. DU-145/CshRNA and DU-145/MUC1shRNA#2 cells (**a**) and MDA-MB-436/tet-MUC1shRNA cells treated with vehicle or DOX for 7 days (**b**) were analyzed for XIST RNA levels at the indicated hours after addition of actinomycin. The results (mean ± SD of three determinations) are expressed as relative XIST RNA levels compared to that obtained for control cells (assigned a value of 1). Indicated are the calculated t1/2 values. **c** DU-145/tet-MUC1shRNA cells treated with vehicle or DOX for 7 days were analyzed for XIST m6A methylation by MeRIP. The results (mean ± SD of three determinations) are expressed as relative XIST m6A levels compared to that obtained for vehicle-treated cells (assigned a value of 1). **d**, **e** Lysates from DU-145/tet-MUC1shRNA cells treated with vehicle of DOX for 7 days were immunoblotted with antibodies against the indicated proteins (left). Signals shown in the immunoblots and in a separate biologic replicate were each scanned in triplicate. The results (mean ± SD of six determinations) are expressed as relative signal intensity compared to that obtained for GAPDH (right). **f** Lysates from DU-145 cells transfected with 30 nM control/ASO or MUC1-C/ASO for 24 h were immunoblotted with antibodies against the indicated proteins. **g** Lysates from DU-145 cells expressing the indicated vectors were treated with vehicle or DOX for 7 days and immunoblotted with antibodies against the indicated protein.

In embryonic development, *XIST* transcription is activated by the lncRNA JPX, which is encoded in the XIC and evicts the CTCF suppressor of XIST expression [34–36]. Here, we sought to understand how MUC1-C increases XIST levels in cancer cells. In contrast to XIST regulation in embryonic development, silencing MUC1-C in DU-145 and MDA-MB-436 cells had no apparent effect on *XIST* transcription (Supplementary Fig. S2a, b). Rather, we found that targeting MUC1-C is associated with decreases in XIST RNA stability in DU-145 (Fig. 2a) and MDA-MB-436 (Fig. 2b) cells, indicating that MUC1-C regulates XIST expression by a post-transcriptional mechanism. XIST is modified by m6A methylation, which decreases XIST stability [37, 38]. We found that silencing MUC1-C increases XIST m6A methylation (Fig. 2c). RBM15 and its paralogue RBM15B bind to the XIST A-repeat region and recruit the WTAP and METTL3/14 components of the m6A methylation complex [2]. Of interest, silencing MUC1-C increased RBM15 and RBM15B expression (Fig. 2d). Analogous to these effects, silencing MUC1-C also increased WTAP and METTL3/14 levels (Fig. 2e). In addressing potential off-target effects, we found that targeting MUC1-C with an ASO similarly increases RBM15/B, WTAP and METTL3/14 levels (Fig. 2f). As confirmation of these results, rescue of MUC1-C silencing with Flag-MUC1-CD abrogated the increases in RBM15/B, WTAP and METTL3/14 (Fig. 2g). Moreover, silencing MUC1-C in MDA-MB-436 cells increased expression of RBM15, WTAP and METTL3/14 (Supplementary Fig. S2c). These findings indicated that MUC1-C increases XIST stability by suppressing (i) RBM15/B, WTAP, and METTL3/14 expression, and (ii) XIST m6A methylation in male and female cancer cells.

### MUC1-C regulates the YTHDF2-CNOT1 deadenylase complex

**Fig. 3:**
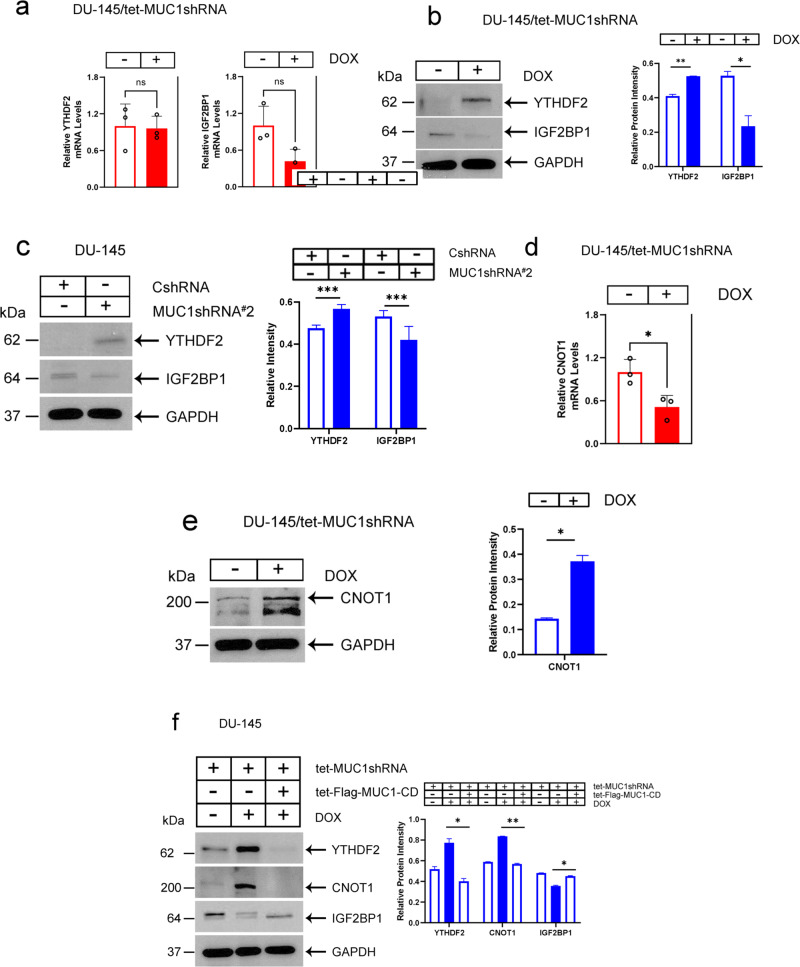
MUC1-C suppresses expression of the YTHDF2 and CNOT1 proteins that destabilize m6A-containing RNAs. **a** DU-145/tet-MUC1shRNA cells treated with vehicle or DOX for 7 days were analyzed for expression of the indicated genes. The results (mean ± SD of three determinations) are expressed as relative mRNA levels compared to that obtained for vehicle-treated cells (assigned a value of 1). Lysates from DU-145/tet-MUC1shRNA cells treated with vehicle or DOX for 7 days (**b**) and DU-145/CshRNA and DU-145/MUC1shRNA#2 (**c**) cells were immunoblotted with antibodies against the indicated proteins. **d** DU-145/tet-MUC1shRNA cells treated with vehicle or DOX for 7 days were analyzed for CNOT1 mRNA levels. The results (mean ± SD of three determinations) are expressed as relative transcript levels compared to that obtained for vehicle-treated cells (assigned a value of 1). **e** Lysates from DU-145/tet-MUC1shRNA cells treated with vehicle or DOX for 7 days were immunoblotted with antibodies against the indicated proteins. **f** Lysates from DU-145 cells expressing the indicated vectors were treated with vehicle or DOX for 7 days and immunoblotted with antibodies against the indicated proteins.

The YTHDF2 “reader” recognizes m6A sites and contributes to RNA decay [39]. In this way, m6A-methylated XIST is recognized by YTHDF2 in promoting XIST degradation [38]. By contrast, the IGF2BP1 “reader” stabilizes m6A-containing RNAs [40, 41]. We found that silencing MUC1-C in DU-145 cells has no significant effect on YTHDF2 and IGF2BP1 transcripts (Fig. 3a). Further analysis demonstrated that silencing MUC1-C regulates YTHDF2 levels and expression of a IGF2BP1 doublet that may be related to post-translational ubiquitination (Fig. 3b). These results were confirmed in DU-145/MUC1shRNA#2 (Fig. 3c) and in MDA-MB-436/tet-MUC1shRNA cells (Supplementary Fig. S3a, b), indicating that MUC1-C regulates YTHDF2 expression by a post-transcriptional mechanism. YTHDF2 interacts with the CNOT1 subunit of the CCR4-NOT deadenylase complex [42]. Silencing MUC1-C in DU-145 cells decreased CNOT1 transcripts (Fig. 3d) and increased CNOT1 protein levels (Fig. 3e). These effects were confirmed in MDA-MB-436 cells (Supplementary Fig. S3c, d), indicating that, like YTHDF2, MUC1-C regulates CNOT1 by a post-transcriptional mechanism. As a control, the effects of MUC1-C silencing on YTHDF2, IGF2BP1 and CNOT1 were reversed by rescue with MUC1-CD (Fig. 3f). These findings indicated that MUC1-C (i) suppresses the YTHDF2 and CNOT1 proteins that destabilize XIST and (ii) upregulates IGF2BP1, which stabilizes m6A-containing RNAs.

### MUC1-C regulates TDP-43 expression

**Fig. 4:**
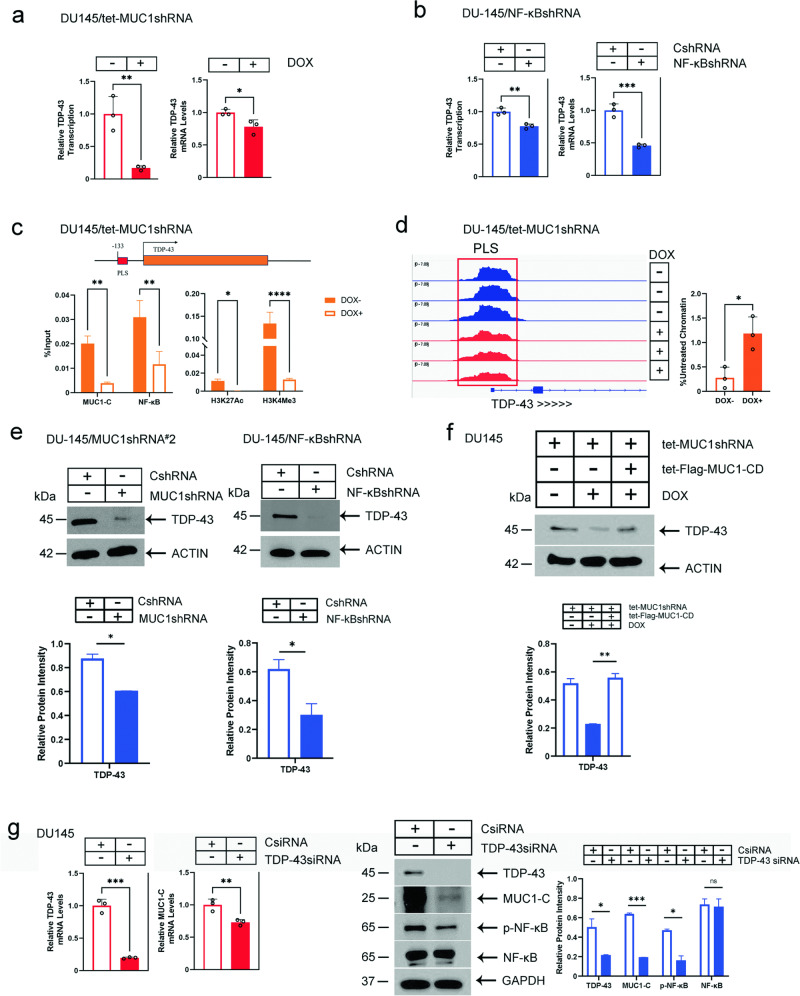
MUC1-C regulates expression of the TDP-43 XIST RBP. **a** DU-145/tet-MUC1shRNA cells treated with vehicle or DOX for 7 days were analyzed for *TDP-43* gene transcription (left) and TDP-43 transcripts (right). The results (mean ± SD of three determinations) are expressed as relative levels compared to that obtained for vehicle-treated cells (assigned a value of 1). **b** DU-145/CshRNA and DU-145/NF-κBshRNA cells were analyzed for *TDP-43* gene transcription (left) and TDP-43 transcripts (right). The results (mean ± SD of three determinations) are expressed as relative levels compared to that obtained for vehicle-treated cells (assigned a value of 1). **c** Schema of the *TDP-43* gene with highlighting of the PLS region. Soluble chromatin from DU-145/tet-MUC1shRNA cells treated with vehicle of DOX for 7 days was precipitated with anti-MUC1-C, anti-NF-κB, anti-H3K27ac, H3K4me1 and H3K4me3. The DNA samples were amplified by qPCR with primers for the *TDP-43* PLS region. The results (mean ± SD of 3 determinations) are expressed as percent input. **d**. Genome browser snapshot of ATAC-seq data from the *TDP-43* PLS in DU-145/tet-MUC1shRNA cells treated with vehicle or DOX for 7 days (left). Chromatin was analyzed for accessibility by nuclease digestion (right). The results (mean ± SD of three determinations) are expressed as % undigested chromatin. **e** Lysates from DU-145/CshRNA and DU-145/MUC1shRNA (left) or DU-145/NF-κBshRNA (right) cells were immunoblotted with antibodies against the indicated proteins. **f** Lysates from DU-145 cells expressing the indicated vectors treated with vehicle or DOX for 7 days were immunoblotted with antibodies against the indicated proteins. **g** DU-145 cells transfected with a control CsiRNA or TDP-43siRNA were analyzed for expression of the indicated genes. The results (mean ± SD of three determinations) are expressed as relative levels compared to that obtained for CsiRNA cells (assigned a value of 1). Lysates were immunoblotted with antibodies against the indicated proteins (right).

The demonstration that MUC1-C regulates the RBM15/B proteins that bind to the XIST A repeats invoked the possibility these findings could extend to other XIST RBPs. In that regard, MUC1-C regulates components of the PRC1/2 complexes that interact with the XIST B/C-repeat arrays [2, 20, 23, 24]. TDP-43 interacts with the XIST E-repeat, plays a role in XCI [2, 43] and is widely involved in the regulation of mRNA alternative processing, transport and stability [44, 45]. TDP-43 is also overexpressed in pan-cancers by unclear mechanisms [44]. Silencing MUC1-C in DU-145 cells suppressed *TDP-43* gene transcription and mRNA levels (Fig. 4a). By contrast, MUC1-C had no significant effect on expression of the *CELF1 and MATR3* genes that encode other XIST E-repeat binding proteins [2] (Supplementary Fig. S4a, b). We also found that, like *MUC1*, NF-κB is necessary for transcription of the *TDP-43* gene and TDP-43 mRNA levels (Fig. 4b). The *TDP-43* gene has a putative NF-κB binding motif in the promoter region, which we found is occupied by MUC1-C and NF-κB (Fig. 4c). Silencing MUC1-C decreased NF-κB occupancy of that region (Fig. 4c). Moreover, silencing MUC1-C was associated with decreases in (i) H3K27ac and H3K4me3 levels (Fig. 4c) and chromatin accessibility of the *TDP-43* promoter region (Fig. 4d). Consistent with these results, silencing MUC1-C and NF-κB decreased TDP-43 protein levels (Fig. 4e), which were rescued with MUC1-CD (Fig. 4f). In addition, we found that, as shown for MUC1-C and NF-κB, silencing XIST decreases TDP-43 expression (Supplementary Fig. S4c). In support of an auto-regulatory loop, TDP-43 was also necessary for expression of MUC1-C and p-NF-κB (Fig. 4g).

### MUC1-C and XIST function in an auto-inductive pathway that regulates common gene signatures

**Fig. 5:**
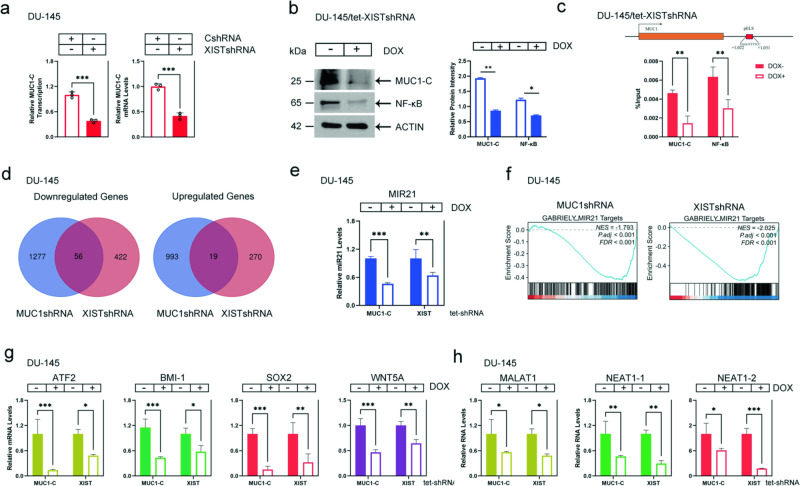
MUC1-C and XIST form an auto-inductive pathway. **a** DU-145/CshRNA and DU-145/XISTshRNA cells were analyzed for *MUC1-C* gene transcription and MUC1-C mRNA levels. The results (mean ± SD of three determinations) are expressed as relative levels compared to that obtained for CshRNA cells (assigned a value of 1). **b** Lysates from DU-145/tet-XISTshRNA cells treated with vehicle or DOX for 10 days were immunoblotted with antibodies against the indicated proteins. **c** Schema of the *MUC1* gene with highlighting of the pELS region. Soluble chromatin from DU-145/tet-XISTshRNA cells treated with vehicle of DOX for 7 days was precipitated with anti-MUC1-C and anti-NF-κB. The DNA samples were amplified by qPCR with primers for the *MUC1* pELS region. The results (mean ± SD of 3 determinations) are expressed as percent input. **d** RNA-seq datasets from DU-145/tet-MUC1shRNA and DU-145/tet-XISTshRNA cells treated with vehicle or DOX for 7 days were analyzed for shared vs unshared downregulated and upregulated genes. **e** DU-145/tet-MUC1shRNA and DU-145/tet-XISTshRNA cells treated with vehicle or DOX for 7 days were analyzed for miR-21 RNA levels. The results (mean ± SD of three determinations) are expressed as relative levels compared to that obtained for vehicle-treated cells (assigned a value of 1). **f** GSEA of the DOX-treated DU-145/tet-MUC1shRNA and DU-145/tet-XISTshRNA RNA-seq datasets using the GABRIELLY MIR21 TARGETS gene signature. DU-145/tet-MUC1shRNA and DU-145/tet-XISTshRNA cells treated with vehicle or DOX for 7 days were analyzed for mRNA (**g**) and lncRNA (**h**) levels. The results (mean ± SD of three determinations) are expressed as relative levels compared to that obtained for vehicle-treated cells (assigned a value of 1).

MUC1-C has been linked to certain effectors through the formation of auto-inductive pathways [31]. To determine if XIST regulates MUC1-C, we silenced XIST with a tet-XISTshRNA (Supplementary Fig. S5a) and found downregulation of *MUC1* gene transcription and mRNA levels (Fig. 5a). Targeting XIST with a second XISTsiRNA similarly downregulated MUC1-C expression (Supplementary Fig. S5b, c). XIST regulates NF-κB activity in the inflammatory response [3, 4]. MUC1-C binds directly to NF-κB p65 and promotes activation of NF-κB target genes [21, 22]. Here, silencing XIST decreased MUC1-C and NF-κB levels (Fig. 5b), indicating that XIST may regulate MUC1-C expression by promoting NF-κB-driven activation of the *MUC1* gene. Analysis of the *MUC1* enhancer region, which includes an NF-κB binding motif [22], demonstrated that silencing XIST decreases MUC1-C and NF-κB occupancy (Fig. 5c). These results in support of a MUC1-C/XIST auto-inductive pathway suggested that MUC1-C and XIST may regulate common sets of genes. To address this notion, we performed RNA-seq on XIST-silenced DU-145 cells and identified 478 downregulated and 289 upregulated genes (Supplementary Fig. S5d). Comparison of this RNA-seq dataset with one derived from MUC1-C-silenced DU-145 cells uncovered 56 and 19 common downregulated and upregulated genes, respectively (Fig. 5d; Supplementary Table S3). MUC1-C and XIST have been linked to the regulation of diverse miRNAs associated with cancer progression [9, 33]. Among common miRNAs identified in in the RNA-seq datasets, we confirmed that MUC1-C and XIST are necessary for expression of miR-21 (Fig. 5e), which was of interest in that miR-21 is overexpressed across pan-cancers and is associated with the regulation of NF-κB and inflammation [46, 47]. By extension, GSEA demonstrated that MUC1-C and XIST significantly regulate the GABRIELLY MIR21 TARGETS gene signature (Fig. 5f; Supplementary Fig. S5d). GSEA also uncovered that MUC1-C and XIST regulate the MIR146A TARGETS gene signature, which like miR-21, miR-146a associates with inflammation and cancer [46, 47]. Moreover, we found that MUC1-C and XIST commonly regulate the TP53 (Supplementary Fig. S5f) and mRNA destabilization (Supplementary Fig. S5g) gene signatures. Focusing here on other selected genes associated with inflammation and stemness, we confirmed that MUC1-C and XIST regulate expression of ATF2 [48], SOX2 [49], BMI1 [20] and WNT5A [50] (Fig. 5g) genes. We also found that the MUC1-C/XIST pathway regulates expression of the NEAT1 and MALAT1 lncRNAs (Fig. 5h), which have been linked to inflammation and cancer progression [51, 52].

### MUC1-C/XIST pathway regulates CSC self-renewal

**Fig. 6:**
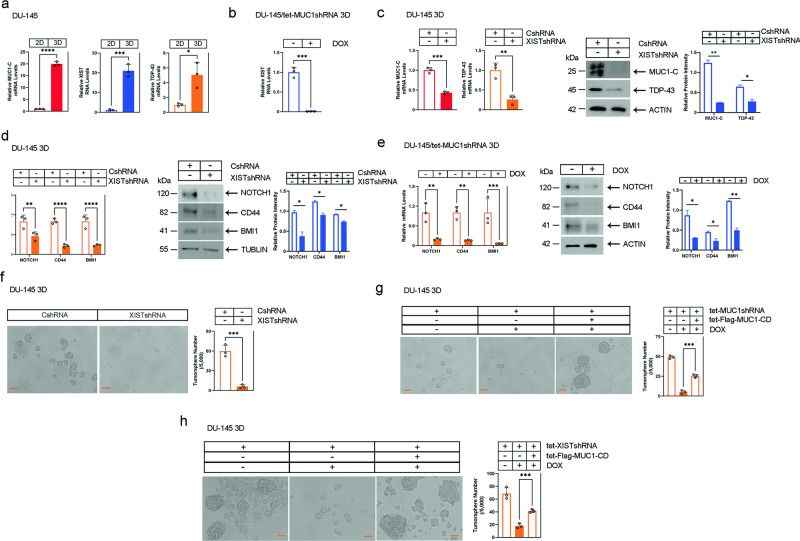
MUC1-C/XIST pathway promotes the CSC state. **a** DU-145 cells grown as 2D monolayers and 3D tumorspheres were analyzed for the indicated RNA levels. The results (mean ± SD of three determinations) are expressed as relative levels compared to that obtained for 2D cells (assigned a value of 1). **b** DU-145 3D/tet-MUC1-CshRNA cells treated with vehicle or DOX for 7 days were analyzed for XIST RNA levels. The results (mean ± SD of three determinations) are expressed as relative levels compared to that obtained for vehicle-treated cells (assigned a value of 1). **c** DU-145 3D/CshRNA and DU-145 3D/XISTshRNA cells were analyzed for the indicated mRNA levels. The results (mean ± SD of three determinations) are expressed as relative mRNA levels compared to that obtained for CshRNA-expressing cells (assigned a value of 1)(left). Lysates were immunoblotted with antibodies against the indicated proteins (right). **d** DU-145 3D/CshRNA and DU-145 3D/XISTshRNA were analyzed for expression of the indicated genes. The results (mean ± SD of three determinations) are expressed as relative mRNA levels compared to that obtained for CshRNA-expressing cells (assigned a value of 1)(left). Lysates were immunoblotted with antibodies against the indicated proteins (right). **e** DU-145 3D/tet-MUC1shRNA cells treated with vehicle or DOX for 7 days were analyzed for expression of the indicated genes. The results (mean ± SD of three determinations) are expressed as relative mRNA levels compared to that obtained for vehicle-treated cells (assigned a value of 1)(left). Lysates were immunoblotted with antibodies against the indicated proteins (right). **f** DU-145 3D/CshRNA and DU-145 3D/XISTshRNA were analyzed for tumorsphere formation. Photomicrographs are shown for the tumorspheres (bar represents 100 μm; left). The results (mean ± SD of three determinations) are expressed as tumorsphere number(right). **g**, **h**. DU-145 3D cells expressing the indicated vectors were treated with vehicle or DOX for 7 days and analyzed for tumorsphere formation. Photomicrographs are shown for the tumorspheres (left). The results (mean ± SD of three determinations) are expressed as tumorsphere number (right).

DU-145 CSCs are dependent on MUC1-C for self-renewal as evidenced by the capacity for tumorsphere formation [25, 26, 28]. Analysis of DU-145 cells grown as 2D monolayers vs 3D tumorspheres demonstrated marked increases in MUC1-C and XIST, as well as TDP-43 expression (Fig. 6a). Silencing MUC1-C in DU-145 3D cells was associated with downregulation of XIST RNA levels (Fig. 6b; Supplementary Fig. S6a). Silencing XIST in DU-145 3D cells also suppressed MUC1-C and TDP-43 expression (Fig. 6c), consistent with activation of the MUC1-C/XIST auto-inductive pathway in CSCs. Moreover, we found that MUC1-C and XIST are necessary for expression of the NOTCH1, CD44, and BMI1 CSC markers (Fig. 6d, e), indicating functional involvement in driving the CSC state. Along these lines and as reported for MUC1-C [25, 26, 28], DU-145 3D cells were also dependent on XIST for tumorsphere formation (Fig. 6f). Rescue of MUC1-C silencing with Flag-MUC1-CD was sufficient to reestablish self-renewal capacity (Fig. 6g). Importantly, rescue of DU-145/XISTshRNA cells with Flag-MUC1-CD also recovered the capacity for self-renewal and markedly increased size of the tumorspheres (Fig. 6h). Moreover, rescue of XIST expression in MUC1-C-silenced DU-145 cells reversed in part loss of tumorsphere formation (Supplementary Fig. S6b, c). These results indicate that MUC1-C-dependent self-renewal is in part conferred by XIST and that other pathways play a role.

## Discussion

**Fig. 7:**
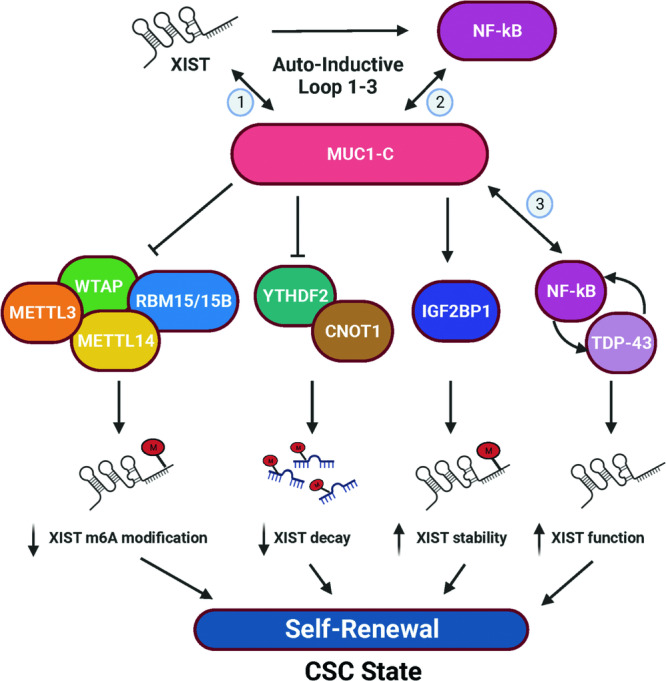
Proposed model for interaction of MUC1-C and XIST in cancer progression. MUC1-C and XIST form an auto-inductive pathway (Loop #1) in cancer cells based on the present results demonstrating that (i) MUC1-C increases XIST RNA levels, and (ii) XIST is necessary for *MUC1* gene transcription and MUC1-C expression. By extension, MUC1-C binds directly to NF-κB in regulating activation of NF-κB target genes, which include *MUC1* in a parallel auto-inductive inflammatory pathway (Loop #2). MUC1-C increases XIST levels by (i) suppressing RBM15/B, WTAP, and METTL3/14 expression and thereby XIST m6A methylation, (ii) downregulating YTHDF2 and CNOT1, which recognize m6A modifications and promote XIST degradation, and (iii) upregulating the IGF2BP1 reader that confers XIST stability. MUC1-C, XIST, and NF-κB are also necessary for expression of TDP-43, which is upregulated in cancer cells, and TDP-43 is necessary for MUC1-C and XIST expression (Loop #3). In support of an inflammatory memory model of cancer progression, we find that the MUC1-C/XIST axis is required for CSC self-renewal capacity.

Transcription of the *XIST* gene is activated by the JPX lncRNA in embryonic development [34–36]; whereas little is known about XIST regulation in somatic cells. XIST is aberrantly upregulated in diverse human cancers from both sexes by unclear mechanisms [9]. Studies in female and male cancer cells have indicated that XIST overexpression is associated with transcription of the active X chromosome [10–13]. The present studies demonstrate that MUC1-C increases XIST expression in male and female cancer cells. Notably, however, MUC1-C had no apparent effect on transcription of the *XIST* gene, in support of distinct regulation from that associated with induction of the active X chromosome. Rather, our results demonstrate that MUC1-C increases XIST stability and thereby XIST lncRNA levels (Fig. 7). The *MUC1* gene is activated in barrier tissues in response to loss of homeostasis [18]. Persistent *MUC1* activation and dysregulation of MUC1-C expression in settings of chronic inflammation contribute to cancer progression [18]. Of interest in this regard, dysregulation of XIST in somatic cells has been linked to the inflammatory response [3–6, 48]. These findings supported potential involvement of MUC1-C in increasing XIST expression and regulating inflammation of somatic cells. This MUC1-C/XIST pathway should, in principle, be reversible with resolution of inflammation; however, if irreversibly established in cancer progression, represents a mechanistic explanation for the upregulation of XIST in pan-cancers.

The XIST transcript is highly modified by m6A methylation [37], which plays a role in regulating lncRNA stability [53]. Consistent with increasing XIST levels, we found that MUC1-C suppresses XIST m6A methylation. RBM15/B recruit the WTAP and METTL3/14 components of the m6A methylation complex to the XIST A repeat region [37]. Interactions of the XIST A repeat with RBM15B and METTL3 are required for XIST m6A methylation and XIST-mediated XCI [37]. In cancer cells, we found that MUC1-C decreases expression of (i) RBM15/B, (ii) WTAP, and (iii) METTL3/14 (Fig. 7). These results supported a model in which MUC1-C suppresses function of the m6A methylation complex in association with downregulation of XIST m6A methylation (Fig. 7). YTHDF2 recognizes m6A sites and contributes to RNA instability by interacting with the CNOT1 subunit of the CCR4-NOT deadenylase complex [39, 42]. We found that MUC1-C suppresses YTHDF2 and CNOT1 expression (Fig. 7). By contrast, MUC1-C increased the expression of IGF2BP1 (Fig. 7), which stabilizes m6A-containing RNAs [40, 41], indicating that MUC1-C coordinates a program of suppressing XIST m6A methylation, recognition and degradation (Fig. 7). Importantly, the effects of targeting MUC1-C on the (i) m6A methylation complex components, (ii) m6A “readers” YTHDF2 and IGF2BP1, and (iii) CNOT1 subunit of the deadenylase complex were rescued by the MUC1-CD cytoplasmic domain, confirming dependence on MUC1-C for their expression (Fig. 7). Of note, IGF2BP1 plays a role in activating NF-κB p65 in chronic inflammation [54–56] and therefore could also contribute to regulation of the MUC1-C/NF-κB auto-inductive pathway and XIST expression.

Our results further demonstrate that MUC1-C and NF-κB are necessary for expression of TDP43, which binds to the XIST E-repeat region where it forms condensates essential for the XIST-independent phase of XCI [43]. TDP-43 is overexpressed in cancer and promotes the dysregulation of mRNA, miRNA, and ncRNA metabolism [44, 57, 58]. We found that MUC1-C and NF-κB occupy the *TDP-43* PLS and that MUC1-C is necessary for (i) NF-κB occupancy and chromatin accessibility of that region and (ii) driving *TDP-43* transcription (Fig. 7). In addition, we identified MUC1-C-dependent increases in H3K27ac and H3K4me3 of the *TDP-43* PLS, consistent with functions of MUC1-C in regulating p300/CBP and the SET1A/WDR5 COMPASS Complex [59]. These findings demonstrate that MUC1-C/NF-κB signaling integrates the regulation of XIST and TDP-43 in cancer cells. Unexpectedly, we found that XIST is necessary for *MUC1* gene activation and MUC1-C expression, in support of an auto-regulatory MUC1-C/XIST pathway. Mechanistically, silencing XIST decreased expression of NF-κB p65, which activates the *MUC1* gene in an auto-inductive MUC1-C/NF-κB circuit [18, 21, 22]. In further support of the MUC1-C/XIST pathway, we found that MUC1-C and XIST regulate common genesets associated with activation of (i) miRNAs, such as miR-21, (ii) lncRNAs NEAT1 and MALAT1, and protein encoding genes that are linked to inflammation and dysregulated in cancer. These findings collectively supported involvement of MUC1-C in driving a previously unrecognized an auto-inductive MUC1-C/XIST pathway in cancer cells (Fig. 7). Our results do not exclude the possibility that the interaction between MUC1-C and XIST is also mediated by the PRC1 and PRC2 repressive complexes, which are activated by MUC1-C and are of importance in XIST-directed deposition of H2AK119ub and H3K27me3 in gene silencing [2, 18].

Our studies performed on male CRPC and female TNBC cancer cells demonstrate that effects of MUC1-C on XIST are consistent across sexes. These results also suggest that MUC1-C co-opts involvement of XIST in post-XCI somatic cells to promote cancer progression (Fig. 7). In support of this notion, we found that the MUC1-C/XIST pathway is necessary for CSC self-renewal. Silencing of MUC1-C and XIST was rescued by MUC1-CD to reestablish self-renewal capacity, in support of the importance of MUC1-CD in driving the CSC state. MUC1-C/CD is a 72 aa disordered protein that is devoid of a kinase function [18]. MUC1-CD is modified by RTKs at the cell membrane and interacts with effectors of the IKK→NF-κB pathway in integrating chronic inflammation with cancer progression. In this way, MUC1-C drives a program of inflammatory memory that is necessary for CSC self-renewal capacity [18, 32]. The present results indicate that the MUC1-C/XIST pathway in somatic cells may also contribute to the establishment of inflammatory memory, which is essential for protection of barrier tissues and has been co-opted by cancer cells in driving the CSC state, DNA damage resistance and immune evasion. Additional evidence supporting involvement of the MUC1-C/XIST pathway in promoting chronic inflammation and inflammatory memory will require further investigation.

## Materials and methods

### Cell culture

Human DU-145 CRPC cells (ATCC) were cultured in RPMI1640 medium (Corning Life Sciences, Corning, NY, USA) containing 10% heat-inactivated FBS. Human NCI-H660 NEPC cells (ATCC) were cultured in RPMI1640 medium containing 5% heat-inactivated FBS, 10 nM β-estradiol (Millipore Sigma), 10 nM hydrocortisone, 1% insulin-transferrin-selenium (ThermoFisher Scientific, Waltham, MA, USA) and 2 mM L-glutamine (ThermoFisher Scientific). Human MDA-MB-436 TNBC cells (ATCC) were cultured in Leibovitz’s L-15 medium (ThermoFisher Scientific) containing 10% heat-inactivated FBS. Human MDA-MB-468 TNBC cells (ATCC) were cultured in Leibovitz’s L-15 medium (ThermoFisher Scientific) containing 10% FBS. Cells were treated with the MUC1-C inhibitor GO-203 [17]. Cells were maintained in culture for 3–4 months. Authentication of the cells was performed by short tandem repeat (STR) analysis. Cells were monitored for mycoplasma contamination using the MycoAlert Mycoplasma Detection Kit (Lonza, Rockland, ME, USA).

### Gene silencing and rescue

MUC1shRNA (MISSION shRNA TRCN0000122938), and a control scrambled shRNA (CshRNA)(Millipore Sigma) were inserted into pLKO-tet-puro (Plasmid #21915; Addgene, Cambridge, MA, USA). XISTshRNA was purchased from GenePharma (Shanghai, China). Control, XIST, and TDP-43 targeted siRNAs (ThermoFisher Scientific) were transfected into cells using Lipofectamine 3000 (ThermoFisher Scientific). DOX-inducible lentiviral shRNA targeting XIST (GE Dharmacon, V3SH11258_245457769) was obtained from Horizon Discovery (Cambridge, UK). DOX-inducible DYRK1A/pTRE3G-FL-hXIST vector was obtained from Addgene (Plasmid #149608; RRID:Addgene_149608). The CshRNA, MUC1shRNA, MUC1shRNA#2 (MISSION shRNA TRCN0000430218), and NF-κBshRNA (MISSION shRNA TRCN0000014687) were produced in HEK293T cells as described [28]. Flag-tagged MUC1-CD [60] was inserted into pInducer20 (Plasmid #44012, Addgene) [61]. Transduced cells were selected for growth in 1–2 μg/ml puromycin or 100 μg/ml geneticin. For inducible gene silencing, cells were treated with 0.1% DMSO as the vehicle control or 500 ng/ml doxycycline (DOX; Millipore Sigma). Cells were transfected with a MUC1/ASO (LG00788741; Qiagen, Hilden, Germany) or a control C/ASO (LG00000001; Qiagen) in the presence of Lipofectamine 3000 Reagent.

### Immunoblot analysis

Whole-cell lysates were prepared in RIPA buffer containing protease inhibitor cocktail (ThermoFisher Scientific). Immunoblotting was performed with anti-MUC1-C (#16564S, 1:1000 dilution; Cell Signaling Technology (CST), Danvers, MA, USA), anti-NF-κB p65 (#8242S, 1:1000 dilution; CST), anti-phospho-NF-κB p65 (#3037s, 1:1000 dilution; CST), anti-GAPDH (5174, 1:1000 dilution; CST), anti-RBM15B (22249-1-AP, 1:1000 dilution; Proteintech, Rosemont, IL, USA), anti-RBM15 (10587-1-AP, 1:5000 dilution; Proteintech), anti-YTHDF2 (24744-1-AP, 1:5000 dilution; Proteintech), anti-CNOT1 (14276-1-AP, 1:1000 dilution; Proteintech), anti-WTAP (#56501, 1:1000 dilution; CST), anti-METTL3 (#96391, 1:1000 dilution; CST), anti-METTL14 (#51104, 1:1000 dilution; CST), anti-IGF2BP1 (22803-1-AP, 1:5000 dilution; Proteintech), anti-TDP-43 (#32654, 1:1000 dilution; CST), anti-NOTCH1 (#3608S, 1:1000 dilution; CST), anti-BMI1 (#6964P, 1:1000 dilution; CST), anti-CD44 (#5640S, 1:1000 dilution; CST), anti-β-actin (A5441; 1:50000 dilution; Sigma, St. Louis, MO, USA) and anti-tubulin (#2144S, 1:1000 dilution; CST). Signals shown in immunoblots and in a separate biologic replicate were each scanned in triplicate. The results (mean ± SD of six determinations) are expressed as relative signal intensity compared to that obtained for GAPDH.

### Quantitative reverse-transcription PCR (qRT-PCR)

Total cellular RNA was isolated using Trizol reagent (ThermoFisher Scientific). cDNAs were synthesized using the High-Capacity cDNA Reverse Transcription Kit (Applied Biosystems, Grand Island, NY, USA). The cDNA samples were amplified as described [62]. Primers used for qRT-PCR are listed in Supplementary Table 1. The primers for MIR21 (#000397) and relative control U6 (#001093) were purchased from ThermoFisher Scientific. The cDNA samples were amplified using TaqMan MicroRNA Reverse Transcription Kit (ThermoFisher Scientific) according to the manufacturer’s protocol.

### Click-iT nascent RNA assay

Nascent RNA labeling with EU was performed using the Click-iT Nascent RNA Capture kit (Invitrogen, Carlsbad, CA, USA) according to the manufacturer’s protocol. Briefly, cells were pulsed with 0.5 mM EU for 24 h. Total RNA was isolated and the nascent transcripts were captured on streptavidin magnetic beads. cDNA synthesis was performed directly on the beads using the High-Capacity cDNA Reverse Transcription Kit followed by analysis with qRT-PCR.

### RNA Stability Assay

Cells were incubated with 5 μg/ml actinomycin D (11421, Cayman, Ann Arbor, MI, USA) for 0, 3, and 6 h. Total RNA was analyzed by qRT-PCR.

### N6-methyladenosine-RNA immunoprecipitation–quantitative polymerase chain reaction m6A RNA enrichment (MeRIP)

MeRIP experiments were performed using the EpiQuik CUT&RUN m6A RNA Enrichment Kit (#P-9018-24, Epigentek, New York, USA) according to the manufacturer’s protocol. Briefly, RNA containing target m6A-modified regions were cleaved into fragments pulled down with beads bound to an m6A capture antibody. The enriched RNA was transcribed to cDNA using the Superscript VILO cDNA synthesis kit (Invitrogen) followed by analysis with qRT-PCR.

### Chromatin immunoprecipitation (ChIP)

ChIP was performed on cells crosslinked with 1% formaldehyde for 5 min at 37 °C, quenched with 2 M glycine, washed with PBS, and then sonicated in a Covaris E220 sonicator to generate 300–600 bp DNA fragments. Immunoprecipitation was performed using a control IgG (3900S, CST) and antibodies against MUC1-C (#16564S, CST), NF-κB p65 (#ab16502, Abcam), H3K27ac (#ab4729, RRID:AB_2118291, Abcam), and H3K4me3 (#ab8580; RRID:AB_306649, Abcam). Precipitated DNAs were detected by PCR using primers listed in Supplementary Table S2. Quantitation was performed on immunoprecipitated DNA using SYBR-green and the CFX384 real-time PCR machine (Bio-Rad, Berkeley, CA, USA). Data are reported as a percentage of input DNA for each sample.

### RNA-seq analysis

Total RNA from cells cultured in triplicates was isolated using Trizol reagent (Invitrogen) as described [28, 30, 63]. TruSeq Stranded mRNA (Illumina, San Diego, CA, USA) was used for library preparation. Raw sequencing reads were aligned to the human genome (GRCh38.74) using STAR. Raw feature counts were normalized and differential expression analysis using DESeq2 as described [28, 30, 63]. Differential expression rank order for subsequent Gene Set Enrichment Analysis (GSEA) was performed using the fgsea (v1.8.0) package in R.

### ATAC-seq

ATAC-seq libraries were generated from three biologically independent replicates per condition. Library preparation and quality control were performed as described [27, 64]. The raw ATAC-seq data was processed using the pipeline: (https://github.com/macs3-project/genomics-analysis-pipelines). MACS2 was used to generate signal tracks for Integrative Genome Browser (IGV) snapshots as described [27].

### Chromatin accessibility assay

DNAse1 chromatin accessibility assays were performed on chromatin isolated as described [27]. Aliquots of chromatin were left untreated or digested with 3 U/100 μl DNase I (Promega, Madison, WI, USA) for 5 min at room temperature as described [27]. DNA was purified and amplified by qPCR using primers listed in Supplementary Table S2. qPCR results were analyzed according to the formula 100/2^Ct (DNase I) −Ct (no DNase I)^. The data were normalized to input DNA without DNase I treatment as described [27].

### Tumorsphere formation assays

Single-cell suspensions were cultured in MammoCult Human MediumKit (Stemcell Technologies, Cambridge, MA, USA) at a density of 5,000 cells per well of a 6-well ultralow attachment culture plate (Corning) for 10 days as described [27]. Tumorspheres with a diameter >50 microns were counted under an inverted microscope in triplicate wells.

### Statistical analysis

Each experiment was performed with at least three independent biologic replicates. Data are expressed as the mean ± SD. The unpaired Student’s *t*-test was used to examine differences between two groups. A *p* value of <0.05 was considered a statistically significant difference. Graphpad Prism 8 was used for all statistical analyses. Asterisks represent *P ≤ 0.05, **P ≤ 0.01, ***P ≤ 0.001, ****P ≤ 0.0001 with CI = 95%.

### Supplementary information

Supplemental Material

Original Data File

## Data Availability

The RNA-seq data have been deposited in the GEO database under accession codes GSE164141 and GSE203055. Data in this study is available upon reasonable request from the corresponding author at donald_kufe@dfci.harvard.edu.
