# Supplementary material for: XIST and MUC1-C form an auto-regulatory pathway in driving cancer progression
Source: Cell Death Dis. 2024 May 13;15(5):330. doi: 10.1038/s41419-024-06684-9 (PMC11091074; doi:10.1038/s41419-024-06684-9)
Supplement: Supplementary file 1 — Supplemental Material [file 41419_2024_6684_MOESM1_ESM.pdf]

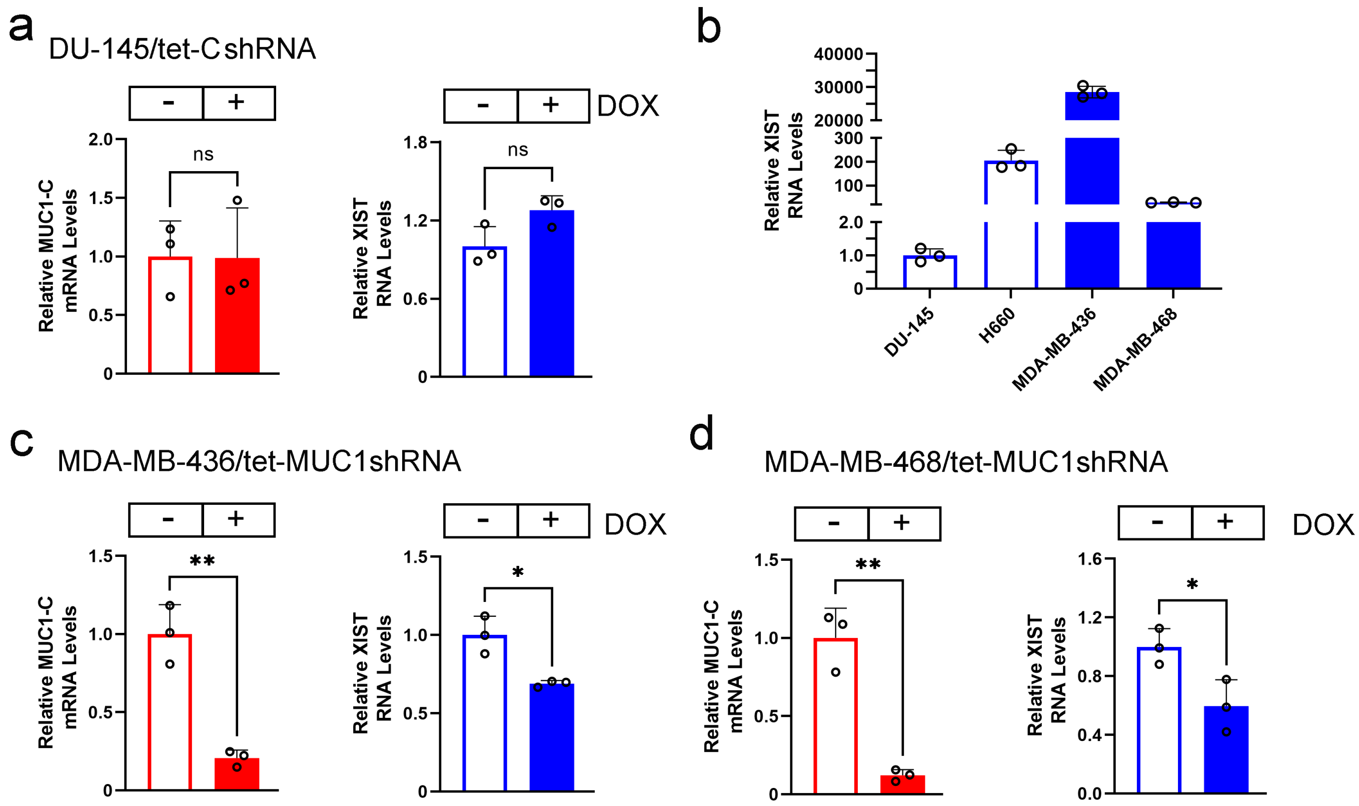

Supplemental Figure S1. MUC1-C upregulates XIST expression. a. DU-145/tet-CshRNA cells treated with vehicle of DOX for 7 days were analyzed for MUC1-C mRNA and XIST RNA levels. The results (mean±SD of three determinations) are expressed as relative transcript levels compared to that obtained for control cells (assigned a value of 1). **b.** The indicated cells were analyzed for XIST RNA levels. The results (mean±SD of three determinations) are expressed as relative transcript levels compared to that obtained for DU-145 cells (assigned a value of 1). **c and d.** MDA-MB-436/tet-MUC1shRNA (**c**) and MDA-MB-468/tet-MUC1shRNA (**d**) cells treated with vehicle of DOX for 7 days were analyzed for MUC1-C mRNA and XIST RNA levels. The results (mean±SD of three determinations) are expressed as relative transcript levels compared to that obtained for vehicle treated cells (assigned a value of 1).

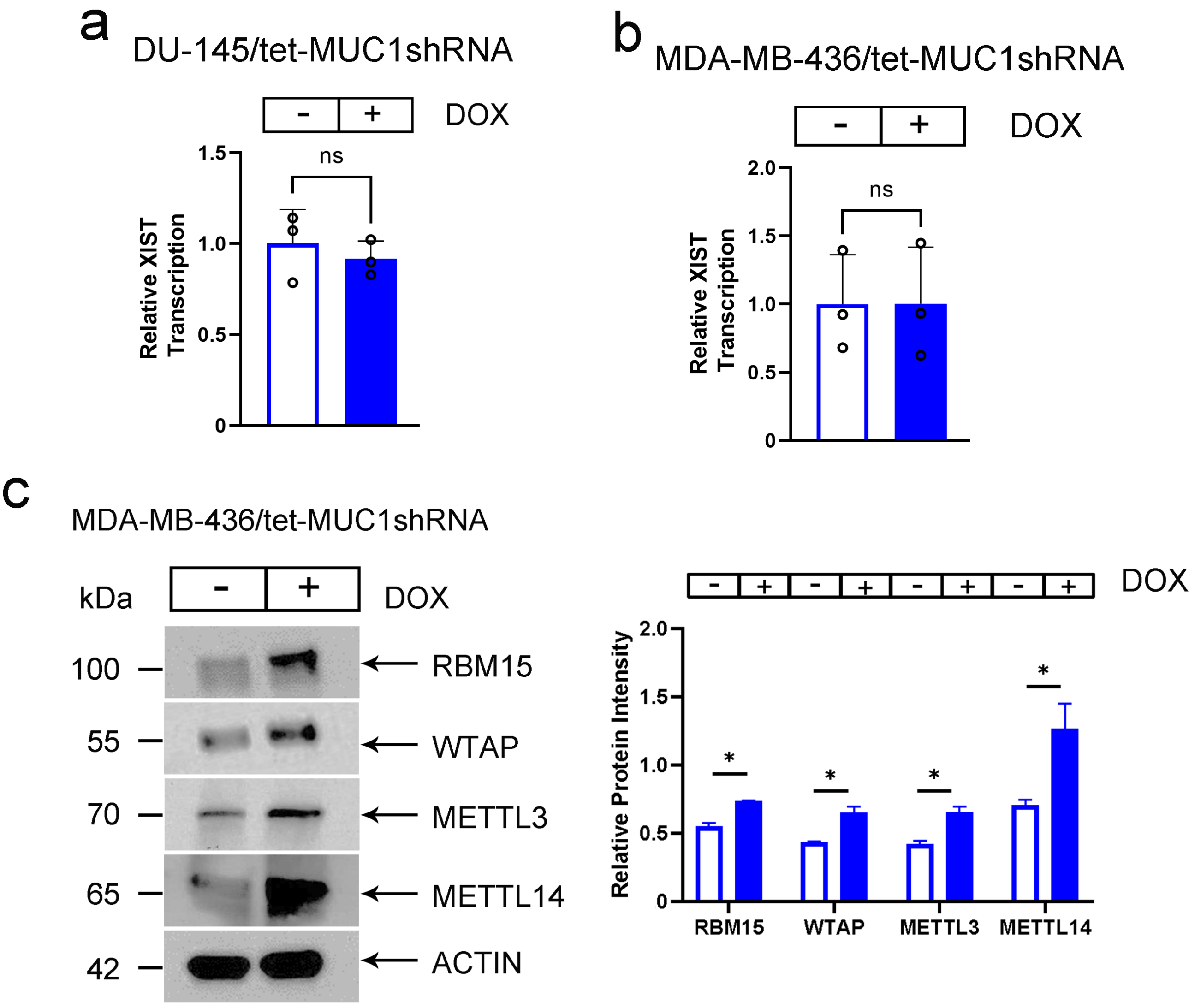

Supplemental Figure S2. MUC1-C regulates RBM15/B, WTAP and METTL3/14 expression. a and b. DU-145/tet-MUC1shRNA (**a**) and MDA-MB-436/tet-MUC1shRNA (**b**) cells treated with vehicle or DOX for 7 days were analyzed for *XIST* transcription. The results (mean±SD of three determinations) are expressed as relative *XIST* transcription compared to that obtained for vehicle-treated cells (assigned a value of 1). **c.** Signals shown in Fig. 2d and in a separate biologic replicate were each scanned in triplicate. The results (mean±SD of six determinations) are expressed as relative signal intensity compared to that obtained for GAPDH. **d.** Signals shown in Fig. 2e and in a separate biologic replicate were each scanned in triplicate. The results (mean±SD of six determinations) are expressed as relative signal intensity compared to that obtained for GAPDH. **e.** Signals shown in Fig. 2f and in a separate biologic replicate were each scanned in triplicate. The results (mean±SD of six determinations) are expressed as relative signal intensity compared to that obtained for GAPDH. **f.** Signals shown in

Fig. 2g and in a separate biologic replicate were each scanned in triplicate. The results (mean±SD of six determinations) are expressed as relative signal intensity compared to that obtained for GAPDH. **g.** Lysates from MDA-MB-436/tet-MUC1shRNA cells treated with vehicle or DOX for 10 days were immunoblotted with antibodies against the indicated proteins. **h.** Signals shown in Supplemental Fig. S2g and in a separate biologic replicate were each scanned in triplicate. The results (mean±SD of six determinations) are expressed as relative signal intensity compared to that obtained for GAPDH.

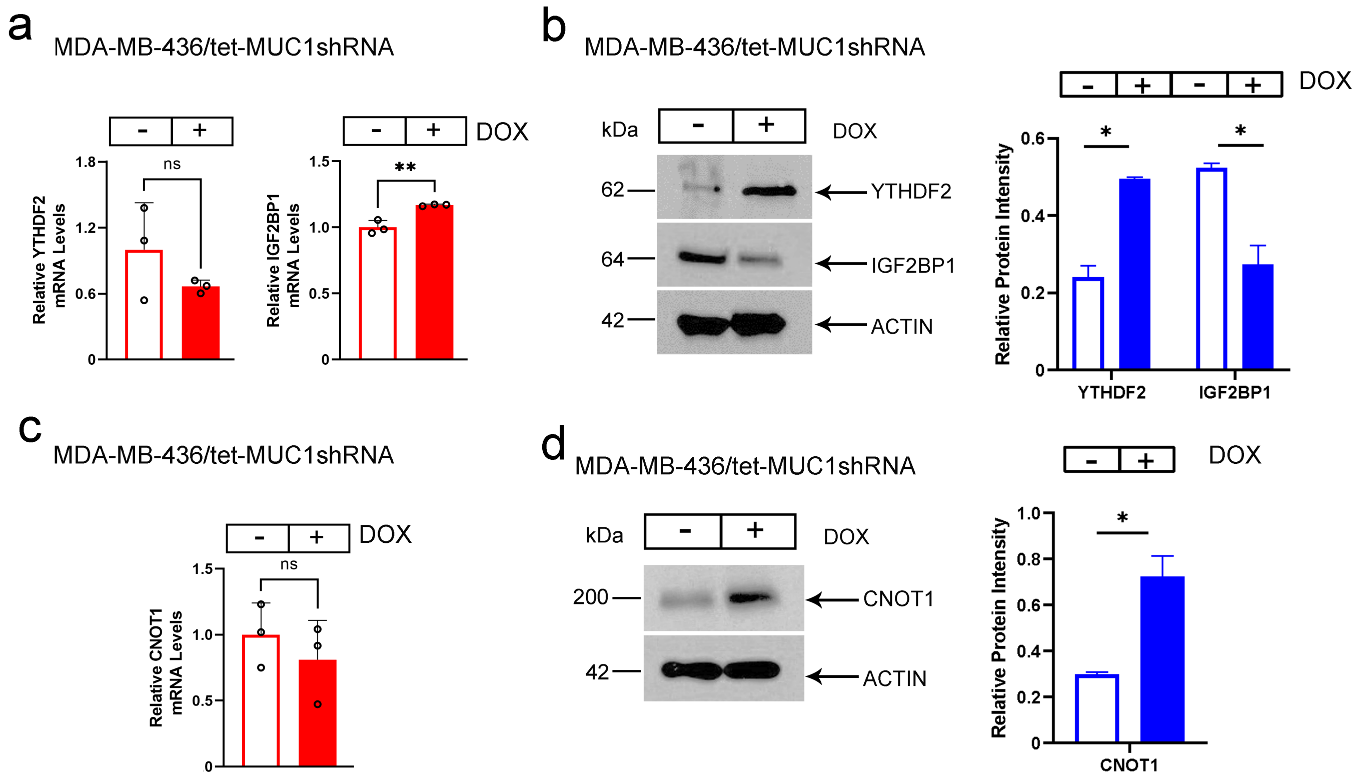

Supplemental Figure S3. Effects of MUC1-C on YTHDF2 and CNOT1 expression. a. MDA-MB-436/tet-MUC1shRNA cells treated with vehicle or DOX for 7 days were analyzed for the indicated mRNA levels. The results (mean±SD of three determinations) are expressed as relative mRNA levels compared to that obtained for vehicle treated cells (assigned a value of 1). **b.** Lysates from MDA-MB-436/tet-MUC1shRNA cells treated with vehicle or DOX for 7 days were immunoblotted with antibodies against the indicated proteins. **c.** MDA-MB-436/tet-MUC1shRNA cells treated with vehicle or DOX for 7 days were analyzed for CNOT1 mRNA levels. The results (mean±SD of three determinations) are expressed as relative mRNA levels compared to that obtained for vehicle treated cells (assigned a value of 1). **d.** Lysates from MDA-MB-436/tet-MUC1shRNA cells treated with vehicle or DOX for 7 days were immunoblotted with antibodies against the indicated proteins.

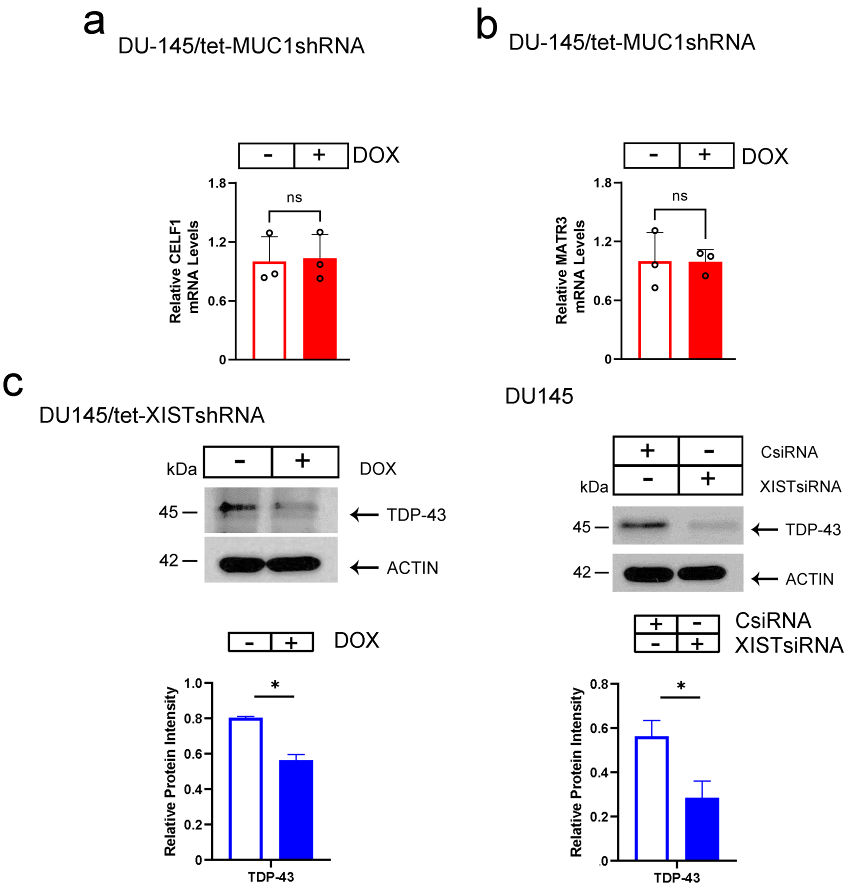

Supplemental Figure S4. Effects of MUC1-C on CELF1, MATR3 and TDP-43 expression. a and b. DU-145/tet-MUC1shRNA cells treated with vehicle or DOX for 7 days were analyzed for CELF1 (**a**) and MATR3 (**b**) mRNA levels. The results (mean±SD of three determinations) are expressed as relative levels compared to that obtained for vehicle treated cells (assigned a value of 1). **c.** Lysates from DU-145/tet-XISTshRNA cells treated with vehicle or DOX for 7 days (left) and DU-145/CsiRNA and DU-145/XISTsiRNA (right) cells were immunoblotted with antibodies against the indicated proteins.

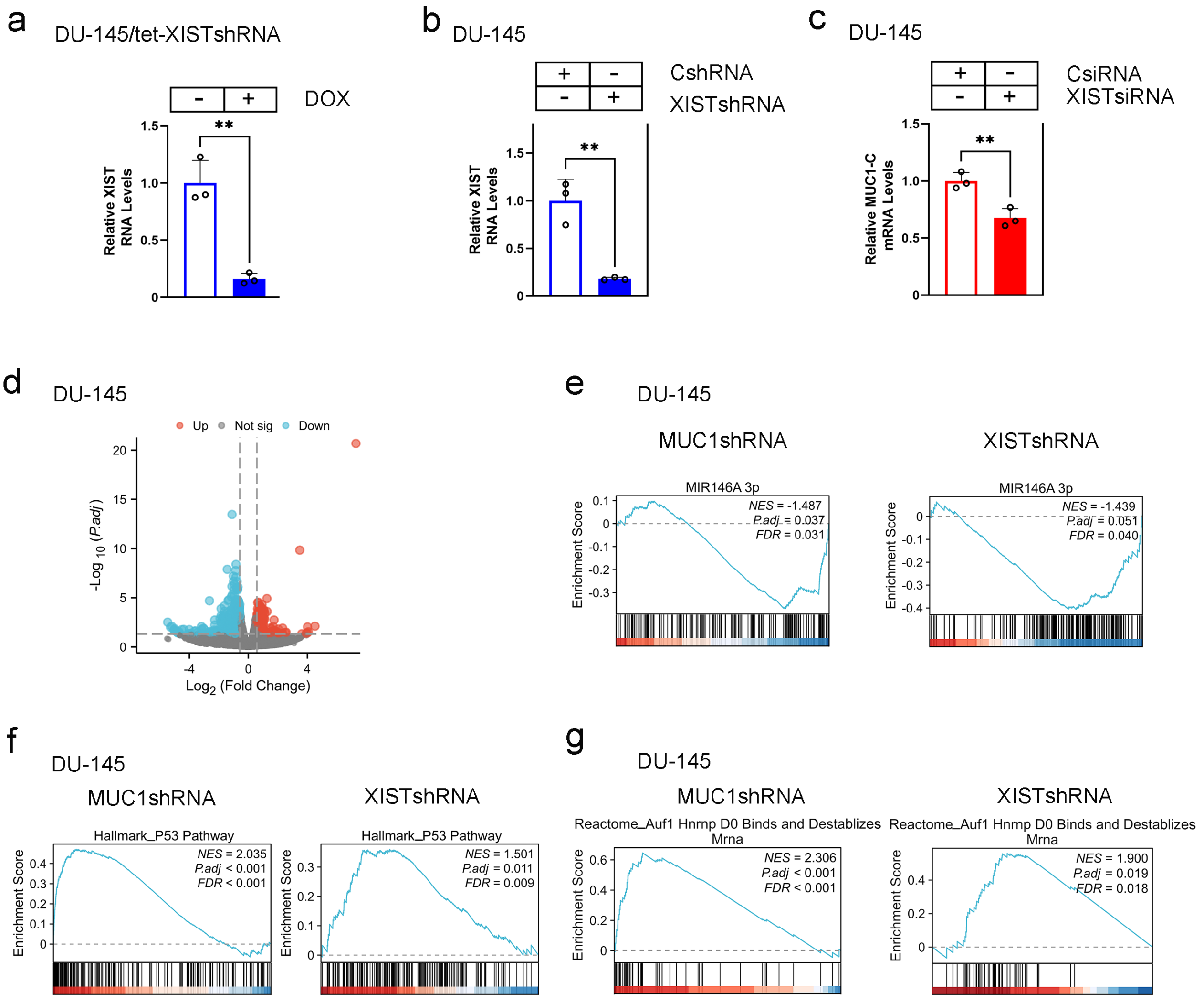

Supplemental Figure S5. Effects of XIST on MUC1-C expression. a. DU-145/tet-XISTshRNA cells treated with vehicle or DOX for 7 days were analyzed for XIST RNA levels. The results (mean±SD of three determinations) are expressed as relative transcript levels compared to that obtained for vehicle-treated cells (assigned a value of 1). **b.** DU-145/CsiRNA and DU-145/XISTsiRNA cells were analyzed for XIST RNA levels. The results (mean±SD of three determinations) are expressed as relative levels compared to that obtained for CsiRNA cells (assigned a value of 1). **c.** DU-145/CsiRNA and DU-145/XISTsiRNA cells were analyzed for MUC1-C expression. The results (mean±SD of three determinations) are expressed as relative MUC1-C mRNA levels compared to that obtained for CsiRNA cells (assigned a value of 1). **d.** RNA-seq was performed in triplicate on DU-145/tet-XISTshRNA cells treated with vehicle or DOX for 7 days. The datasets were analyzed for effects of XIST silencing on down- and up-regulated genes as depicted in the Volcano plot. **e-g.** GSEA of the DOX-treated DU-145/tet-MUC1shRNA and DU-145/tet-XISTshRNA

RNA-seq datasets using the MIR146A-3p (**e**), P53 pathway (**f**) and Auf1 Hnrnp D0 Binds and Destabilizes Mrna (**g**) gene signatures.

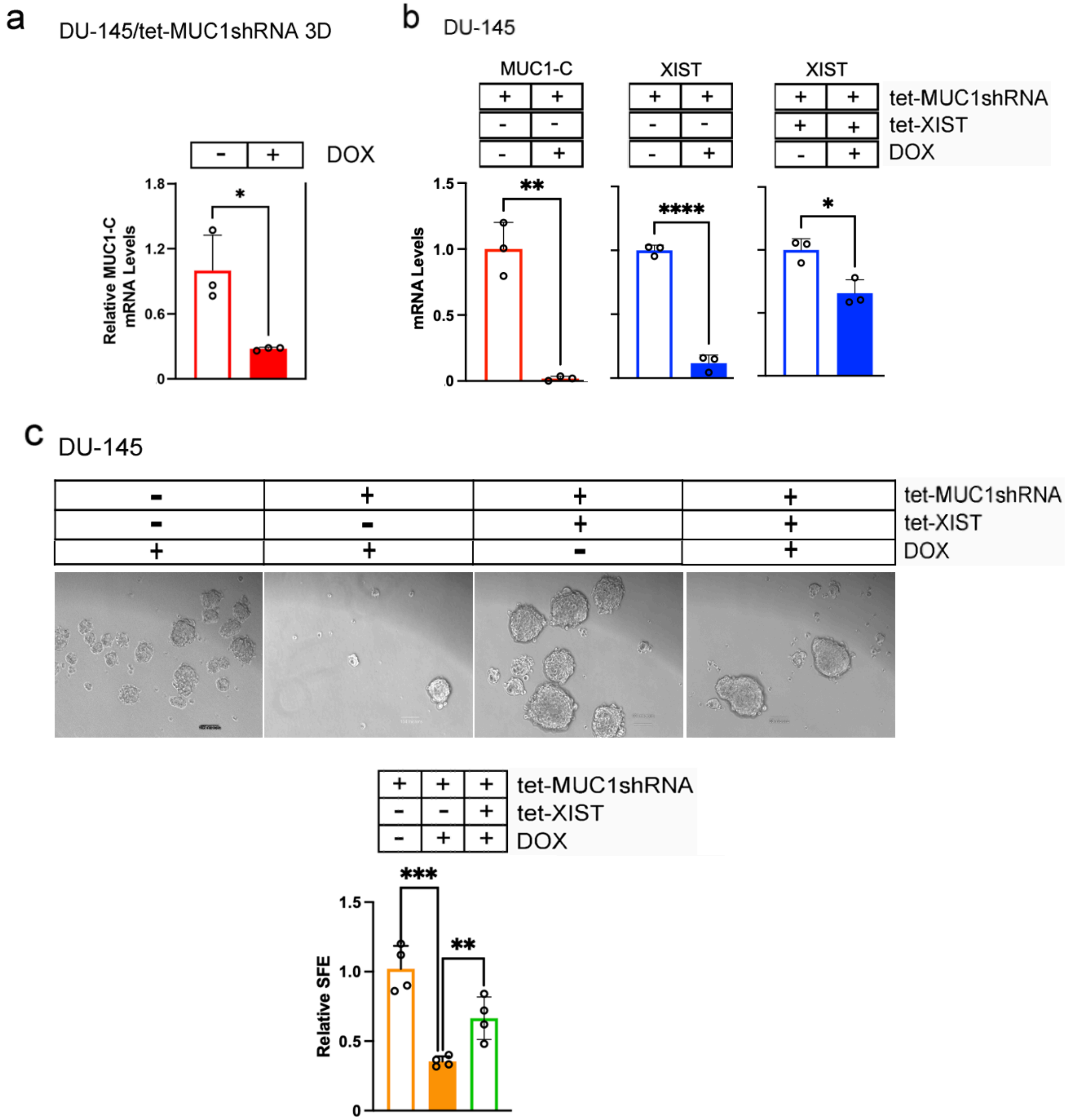

Supplemental Figure S6. Effects of silencing MUC1-C and XIST in DU-145 3D CSCs. a. DU-145/tet-MUC1shRNA 3D cells treated with vehicle or DOX for 7 days were analyzed for MUC1-C expression. The results (mean±SD of three determinations) are expressed as relative mRNA levels compared to that obtained for vehicle treated cells (assigned a value of 1). **b.** DU-145/tet-MUC1shRNA cells expressing the indicated vectors were treated with vehicle or DOX for 7 days and analyzed for MUC1-C and XIST expression. The results (mean±SD of three determinations) are expressed as relative mRNA levels compared to that obtained for vehicle treated cells (assigned a value of 1). **c.** DU-145 cells

expressing the indicated vectors were treated with vehicle or DOX for 7 days and analyzed for tumorsphere formation. Photomicrographs are shown for the tumorspheres. The results (mean±SD of three determinations) are expressed as tumorsphere number.

Supplemental Tables

Supplemental Table S1. Primers used for qRT-PCR analysis.

**MUC1-C**	**FWD**	TACCGATCGTAGCCCCTATG
	**REV**	CTCACCAGCCCAAACAGG
**MUC1-CD**	**FWD**	TGTCAGTGCCGCCGAAAGAA
	**REV**	CTACAAGTTGGCAGAAGTGGCT
**ACTIN**	**FWD**	GATGAGATTGGCATGGCTTT
	**REV**	CACCTTCACCGTTCCAGTTT
**CNOT1**	**FWD**	CATATTGTGAATCGGCACGGT
	**REV**	CCACTGCTTTTACCATCGCCA
**IGF2BP1**	**FWD**	GCGGCCAGTTCTTGGTCAA
	**REV**	TTGGGCACCGAATGTTCAATC
**YTHDF2**	**FWD**	AGCCCCACTTCCTACCAGATG
	**REV**	TGAGAACTGTTATTTCCCCATGC
**TDP-43**	**FWD**	GTGTGGGCTTCGCTACAGG
	**REV**	CAACATACACCAGATTTCCCCAG
**NOTCH1**	**FWD**	GGGCTAACAAAGATATGCAG
	**REV**	ACTGAACCTGACCGTACAGTTGGCAAAGTGGTCCAG
**SOX2**	**FWD**	AGAAGGATAAGTACACGCTGC
	**REV**	TCCAGCCGTTCATGTGC
**BMI1**	**FWD**	GGTACTTCATTGATGCCACAAC
	**REV**	TGCTGGGCATCGTAAGTATC
**CD44**	**FWD**	GGCTTGGAAGAAGATAAAGA
	**REV**	TGCTTGATGTCAGAGTAGAAGT
**XIST-m6A**	**FWD**	CCTTGTGATTAGCACCCTCTAC
	**REV**	TCTCATCCCCTCATATTCCTTTTG
**XIST**	**FWD**	TCATCCCCACTTTTCCCTTC
	**REV**	AATTGTGCACCTAGACTCTCC
**CELF1**	**FWD**	ACATCCGAGTCATGTTCTCTTCG
	**REV**	CATTGCCTTGATAGCCGTCTG
**MATR3**	**FWD**	ATCAATGGAGCAAGTCACAGTC

	**REV**	TGCAACATGAATGGATCACCC
**ATF2**	**FWD**	AATTGAGGAGCCTTCTGTTGTAG
	**REV**	CATCACTGGTAGTAGACTCTGGG
**WNT5A**	**FWD**	ATTCTTGGTGGTCGCTAGG
	**REV**	CTGTCCTTGAGAAAGTCCTG
**MALAT1**	**FWD**	GAAGGAAGGAGCGCTAACG
	**REV**	TACCAACCACTCGCTTTCCC
**NEAT1-1**	**FWD**	CTTCCTCCCTTTAACTTATCCATTCAC
	**REV**	CTCTTCCTCCACCATTACCAACAATAC
**NEAT1-2**	**FWD**	CAGTTAGTTTATCAGTTCTCCCATCCA
	**REV**	GTTGTTGTCGTCACCTTTCAACTCT

Supplemental Table S2. Primers used for ChIP-PCR.

**TDP-43 PLS**	**FWD**	TTGTGCCAGGTACTTTCTGAG
	**REV**	TTTCCTCACCCGCAGAATG
**MUC1-C pELS**	**FWD**	AGCAGAGAATGGAGGGACAA
	**REV**	CACCAGCCAGGTGACTGTAA

Supplemental Table S3. Common genes down-regulated and up-regulated by silencing MUC1-C and XIST.

Common down-regulated genes	Common up-regulated genes
CUBN	BAK1
AHSA2	DNAL4
LGI2	CYPA1
LPGAT1	DDX49
ARL5B	DOHH
ZNF605	HIST1H2BJ
PAG1	HIST1H2BN
N4BP2	PSMD8
AKR1C2	SECTM1
DOCK11	BAD
PBLD	TMEM158
NDNF	CST6
ZC3H6	EIF4EBP1
ZNF137P	PHLDA2
C5	KRT19
EGF	ICAM3
DDX60	EPHB6
JAK2	CLTB
ZNF518A	JOSD2
AGER	
ITGB8	
NPNT	
ZNF780B	
ZNF292	
MAGI2-AS3	
IKZF2	
SESN3	

**ZNF493**	
**NHLRC2**	
**NBEA**	
**ZMAT1**	
**DENND1B**	
**ENOX1**	
**CHML**	
**PGAP1**	
**PCMTD1**	
**SFTPB**	
**ZBTB10**	
**MAP2**	
**TMEM170B**	
**SYCP2**	
**LMO3**	
**BRWD3**	
**RBMS3**	
**DDX60L**	
**ZNF782**	
**AKR1C1**	
**ZNF737**	
**CALD1**	
**ZNF404**	
**ZNF112**	
**TMEM56**	
**TMEM215**	
**PPP4R4**	
**ZNF471**	
**GOLGA8B**	
