# Supplementary material for: XIST and MUC1-C form an auto-regulatory pathway in driving cancer progression
Source: Cell Death Dis. 2024 May 13;15(5):330. doi: 10.1038/s41419-024-06684-9 (PMC11091074; doi:10.1038/s41419-024-06684-9)

Figure 2 d

TET-MUC1shRNA

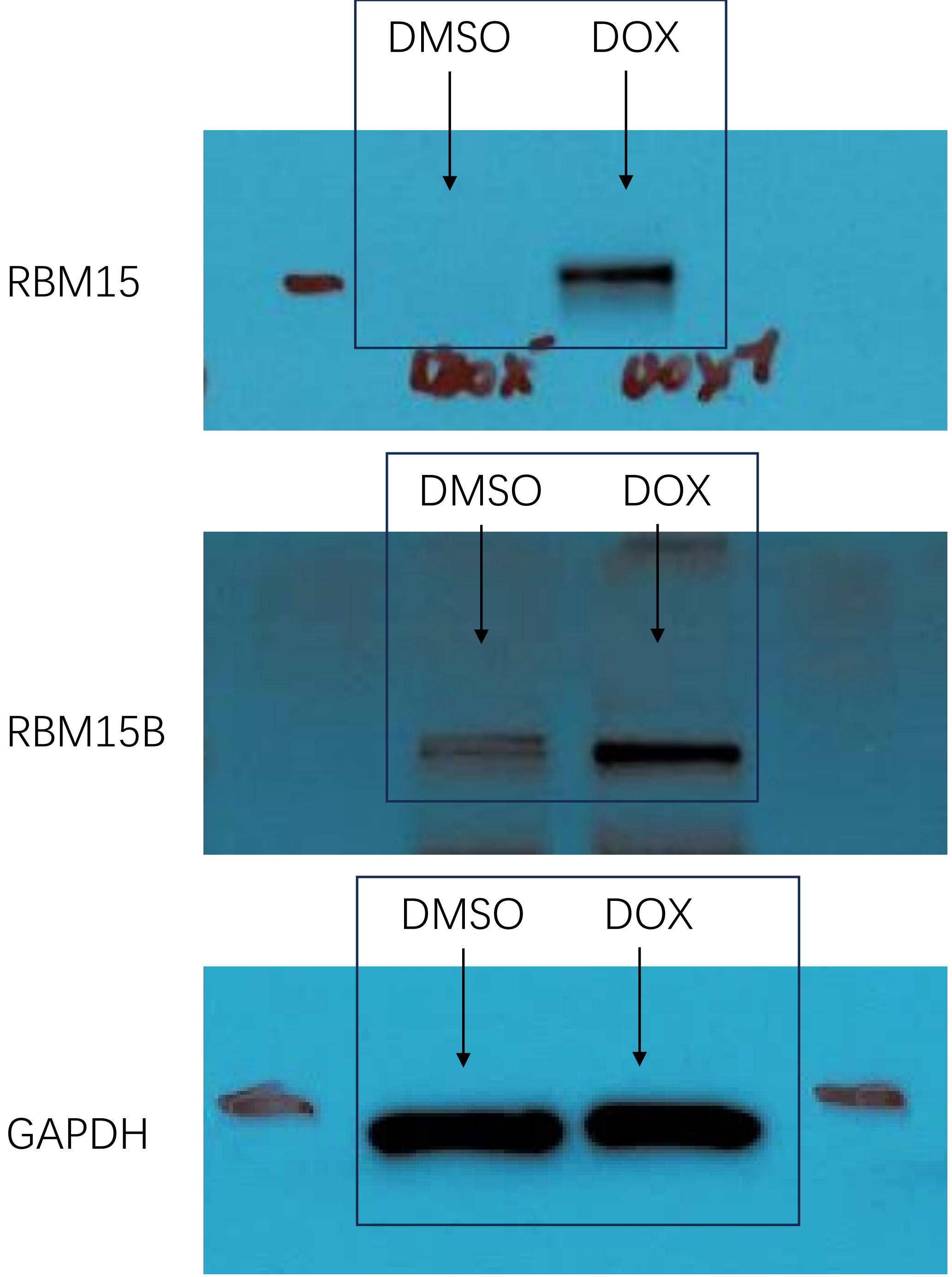

Figure 2 e

TET-MUC1shRNA

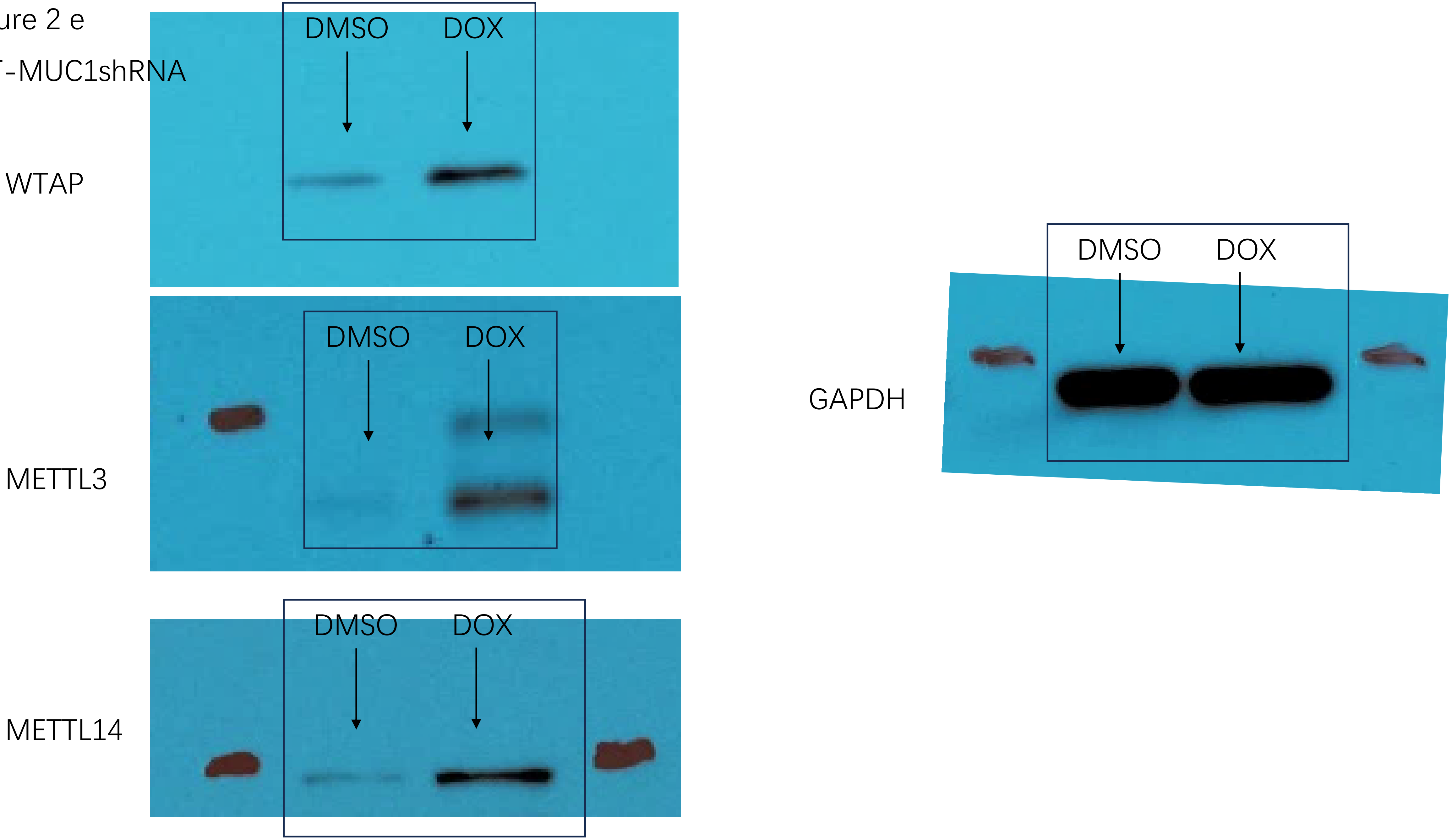

Figure 2 f

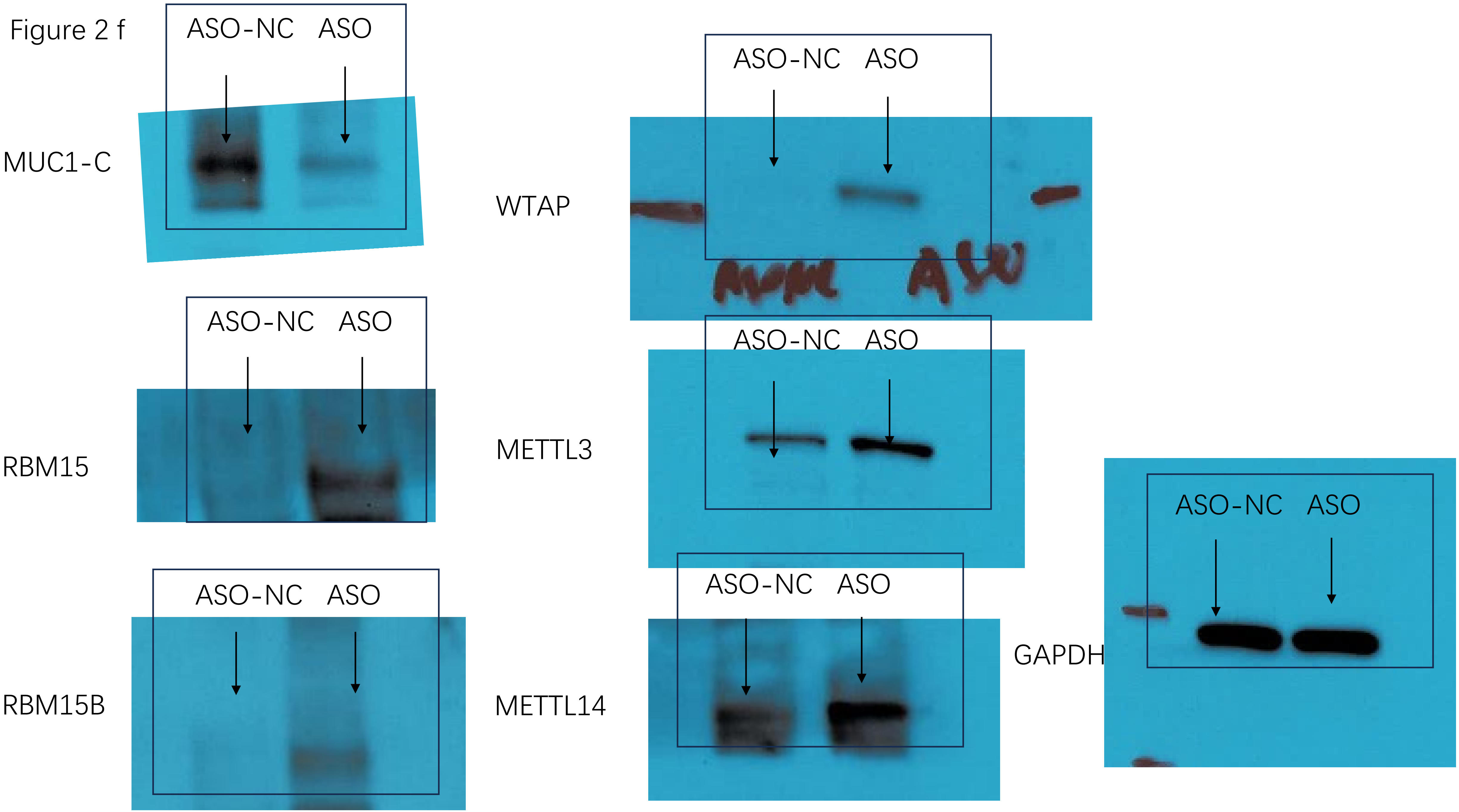
ASO-NC ASO
MUC1-C
ASO-NC ASO
WTAP
ASO-NC ASO
RBM15
ASO-NC ASO
METTL3
ASO-NC ASO
GAPDH
ASO-NC ASO
RBM15B
ASO-NC ASO
METTL14

Figure 2 g

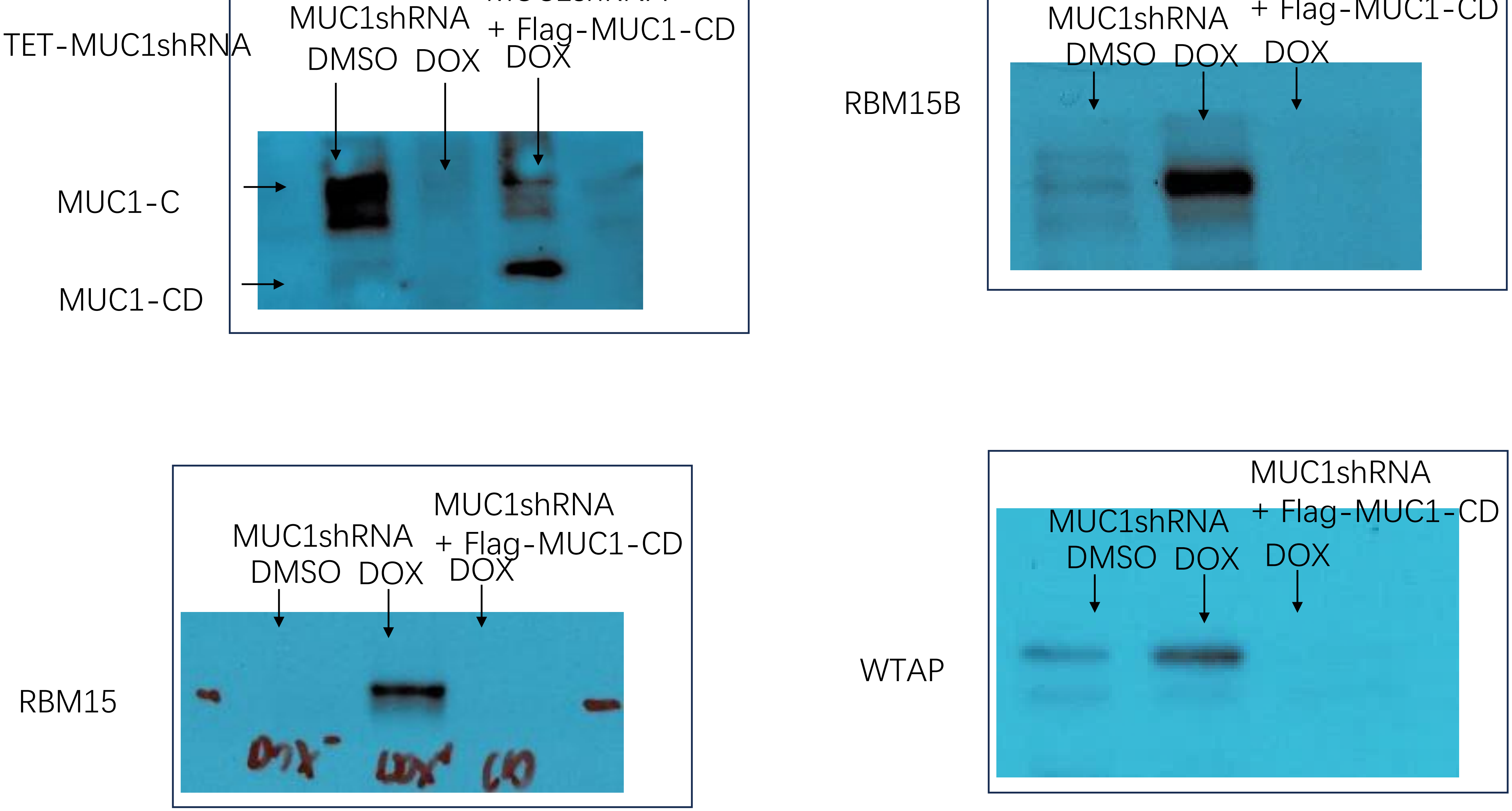
TET-MUC1shRNA
MUC1shRNA
DMSO
DOX
MUC1shRNA
+ Flag-MUC1-CD
DOX
MUC1-C
MUC1-CD
RBM15B
MUC1shRNA
DMSO
DOX
MUC1shRNA
+ Flag-MUC1-CD
DOX
RBM15
MUC1shRNA
DMSO
DOX
MUC1shRNA
+ Flag-MUC1-CD
DOX
WTAP
MUC1shRNA
DMSO
DOX
MUC1shRNA
+ Flag-MUC1-CD
DOX

Figure 2 g

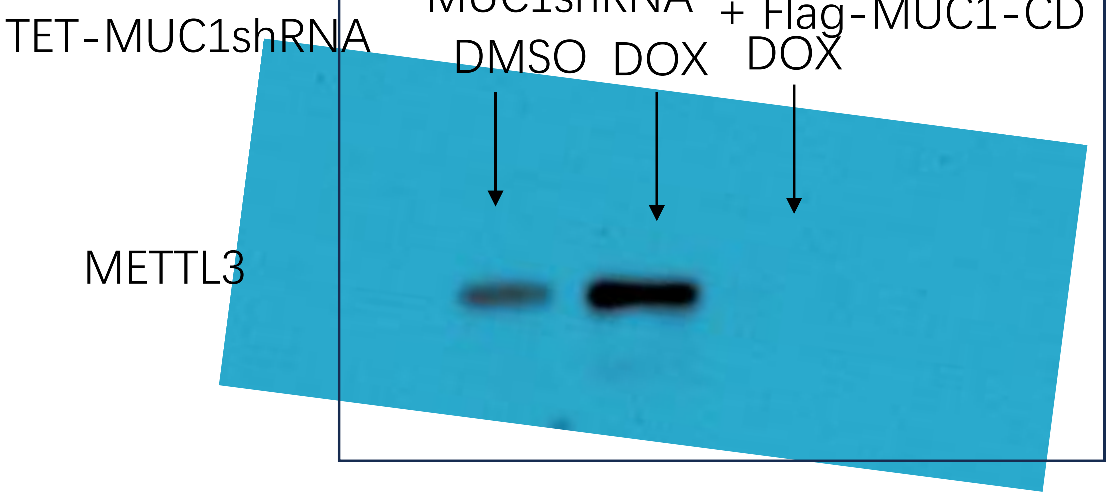
TET-MUC1shRNA
MUC1shRNA
MUC1shRNA
+ Flag-MUC1-CD
DMSO
DOX
DOX
METTL3

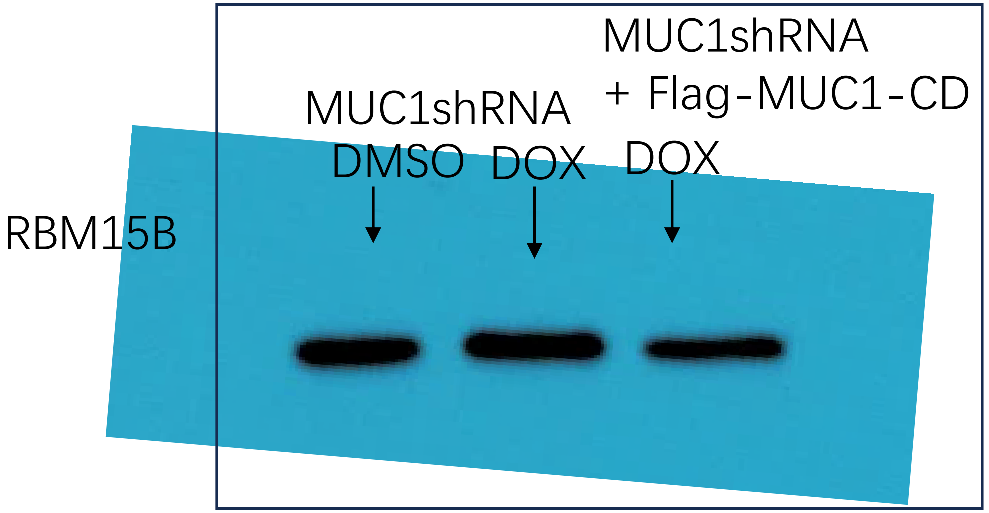
RBM15B
MUC1shRNA
MUC1shRNA
+ Flag-MUC1-CD
DMSO
DOX
DOX

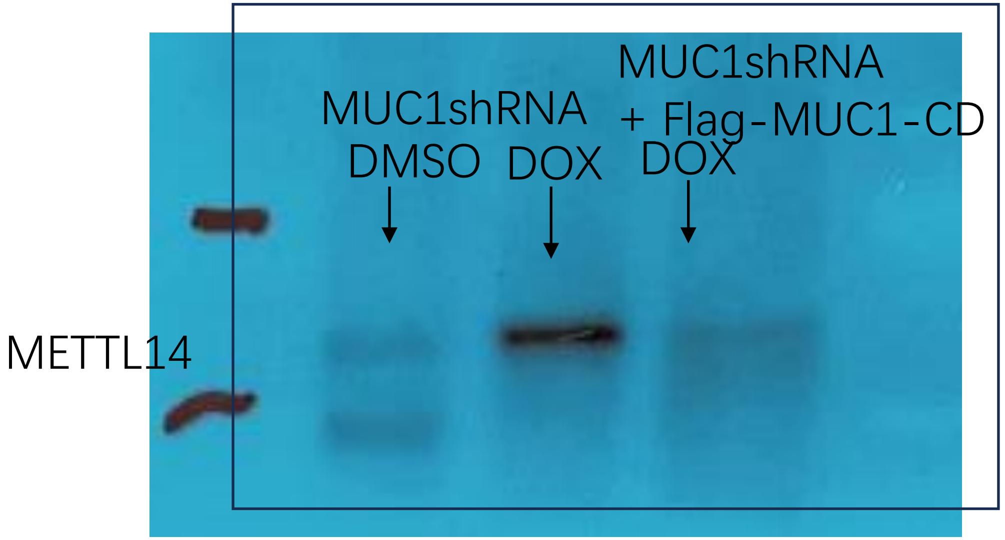
METTL14
MUC1shRNA
MUC1shRNA
+ Flag-MUC1-CD
DMSO
DOX
DOX

Figure S2 c

TET-MUC1shRNA

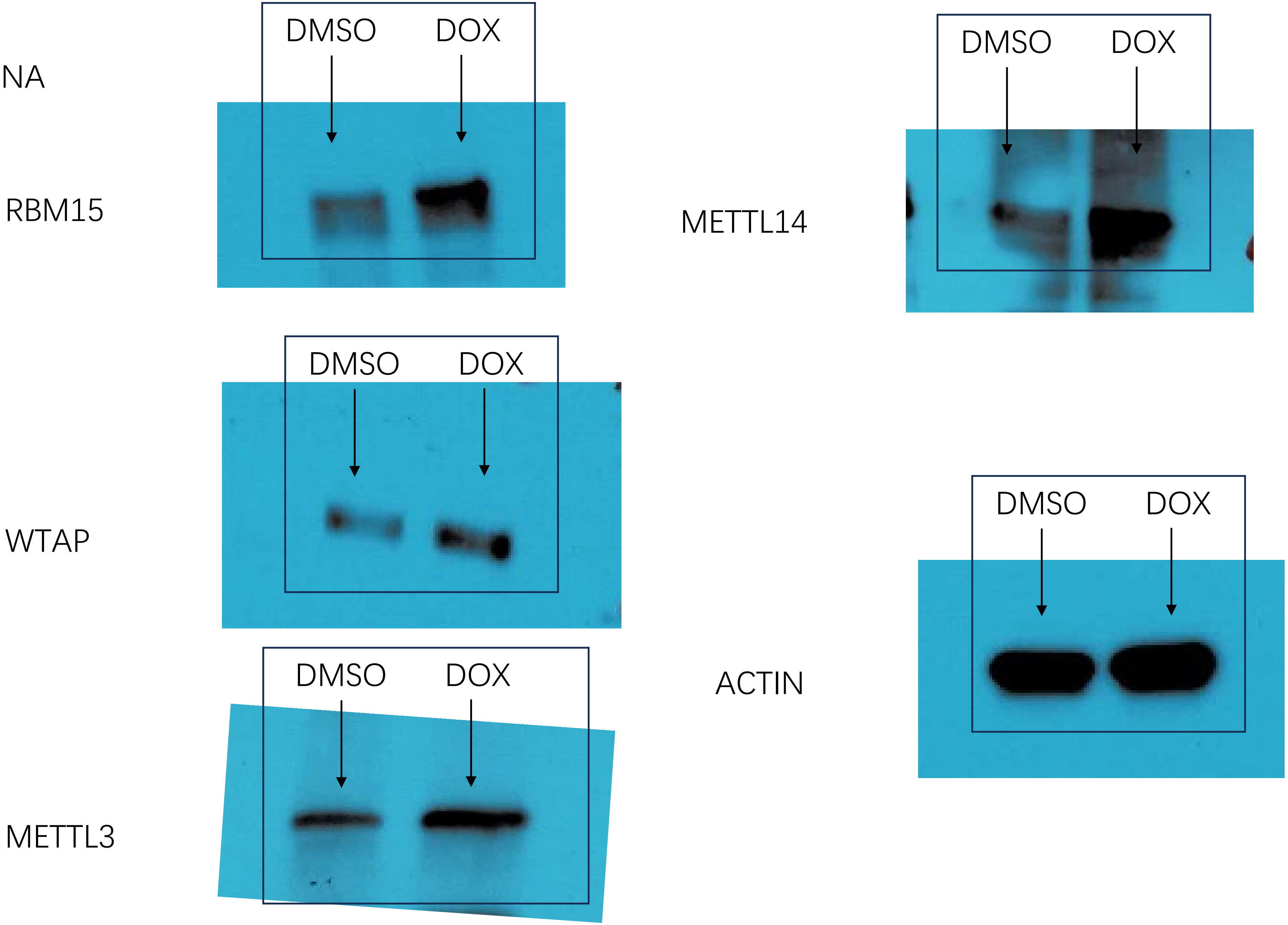

Figure 3 b

TET-MUC1shRNA

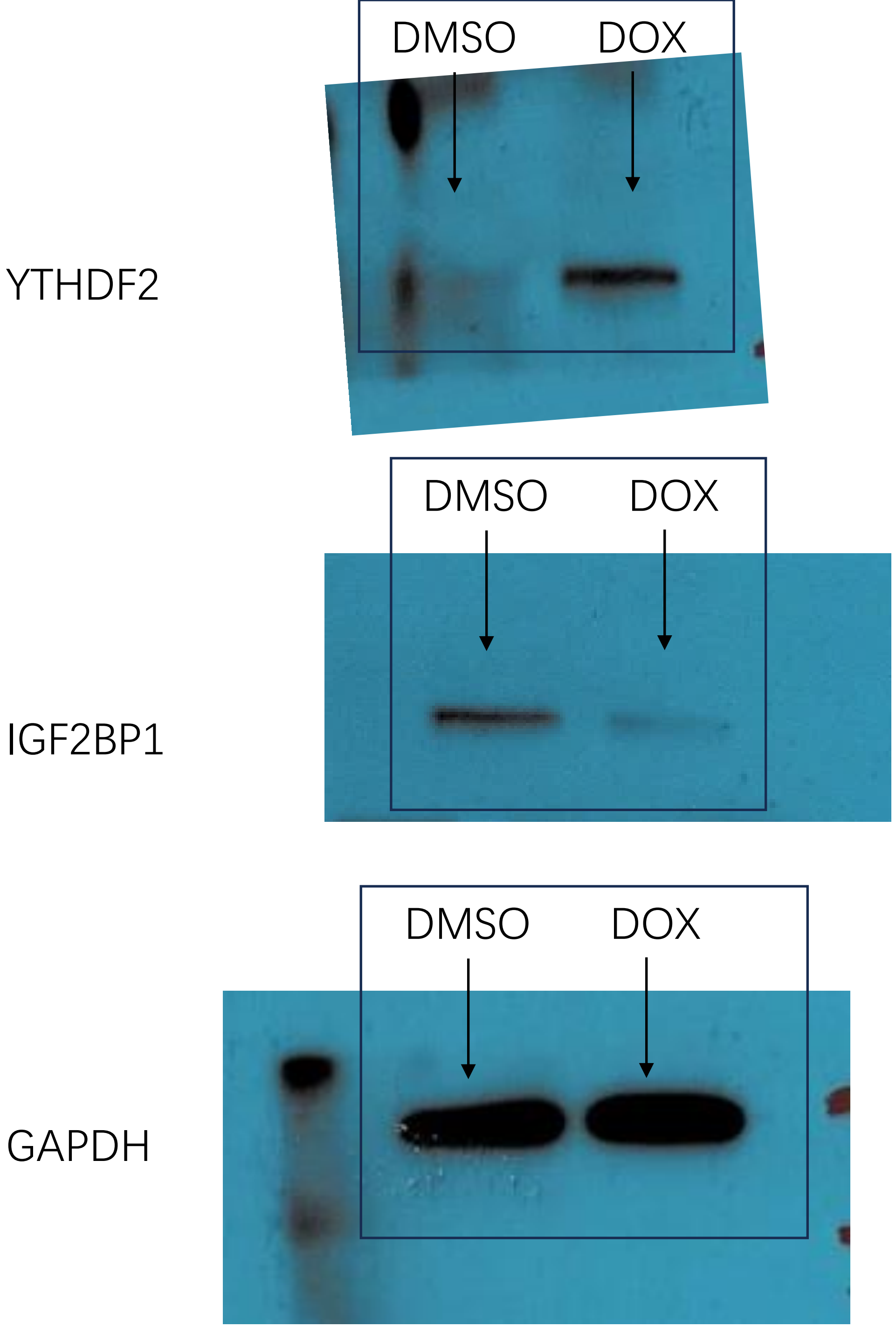

Figure 3 c

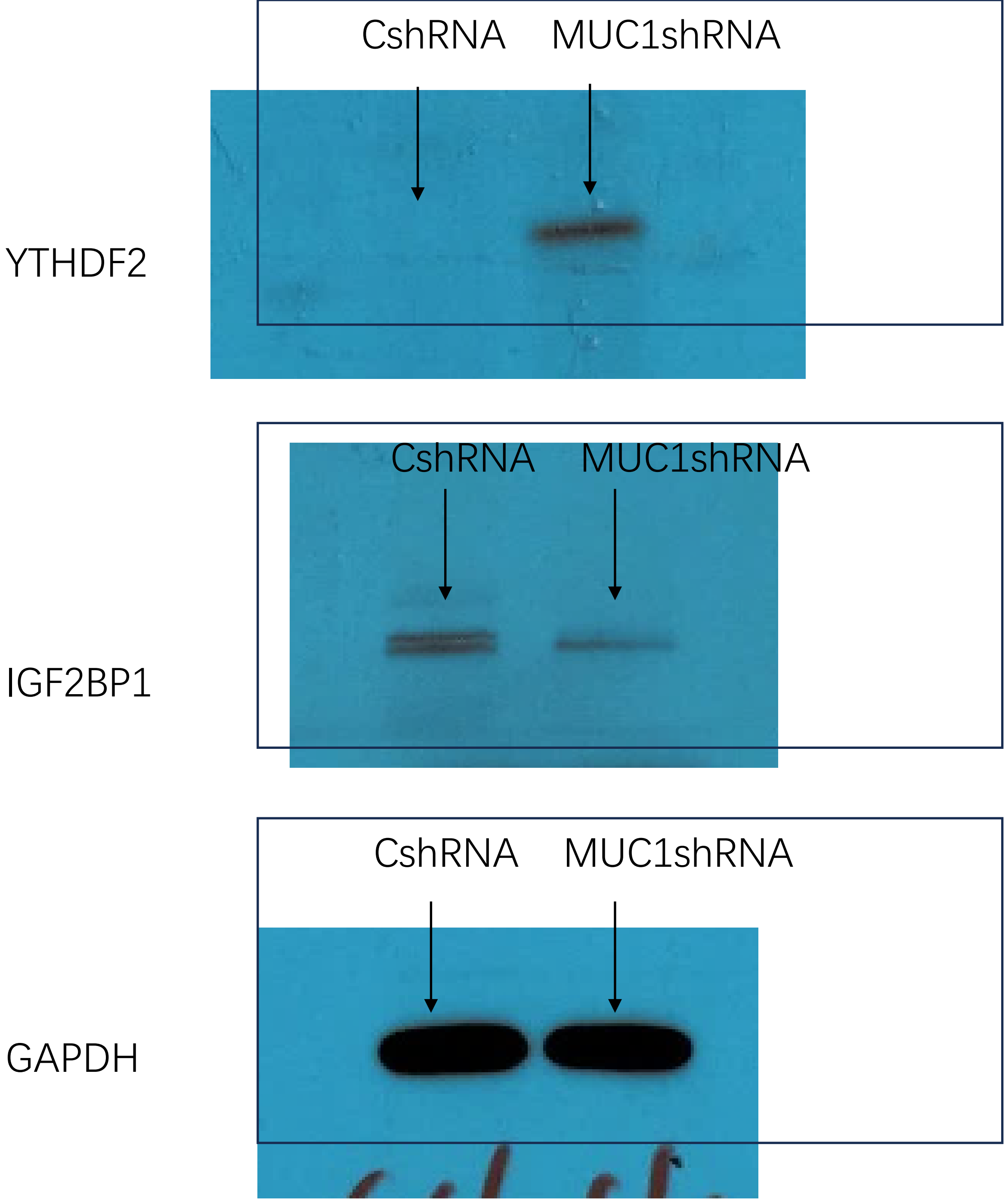
CshRNA
MUC1shRNA
YTHDF2
CshRNA
MUC1shRNA
IGF2BP1
CshRNA
MUC1shRNA
GAPDH

Figure 3 e

TET-MUC1shRNA

CNOT1

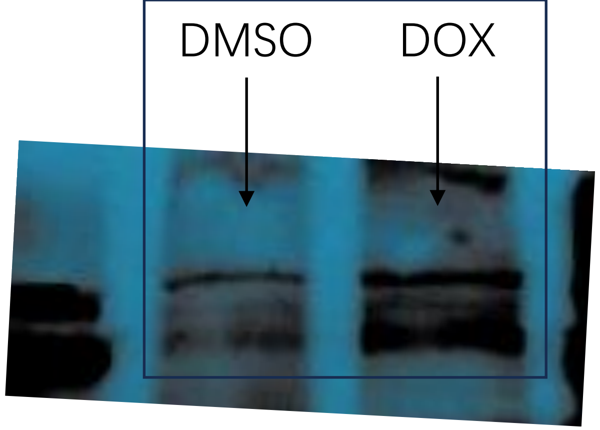

GAPDH

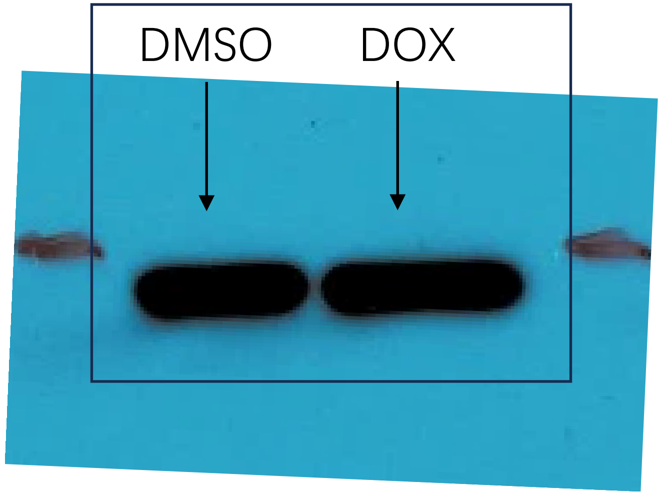

Figure 3 f

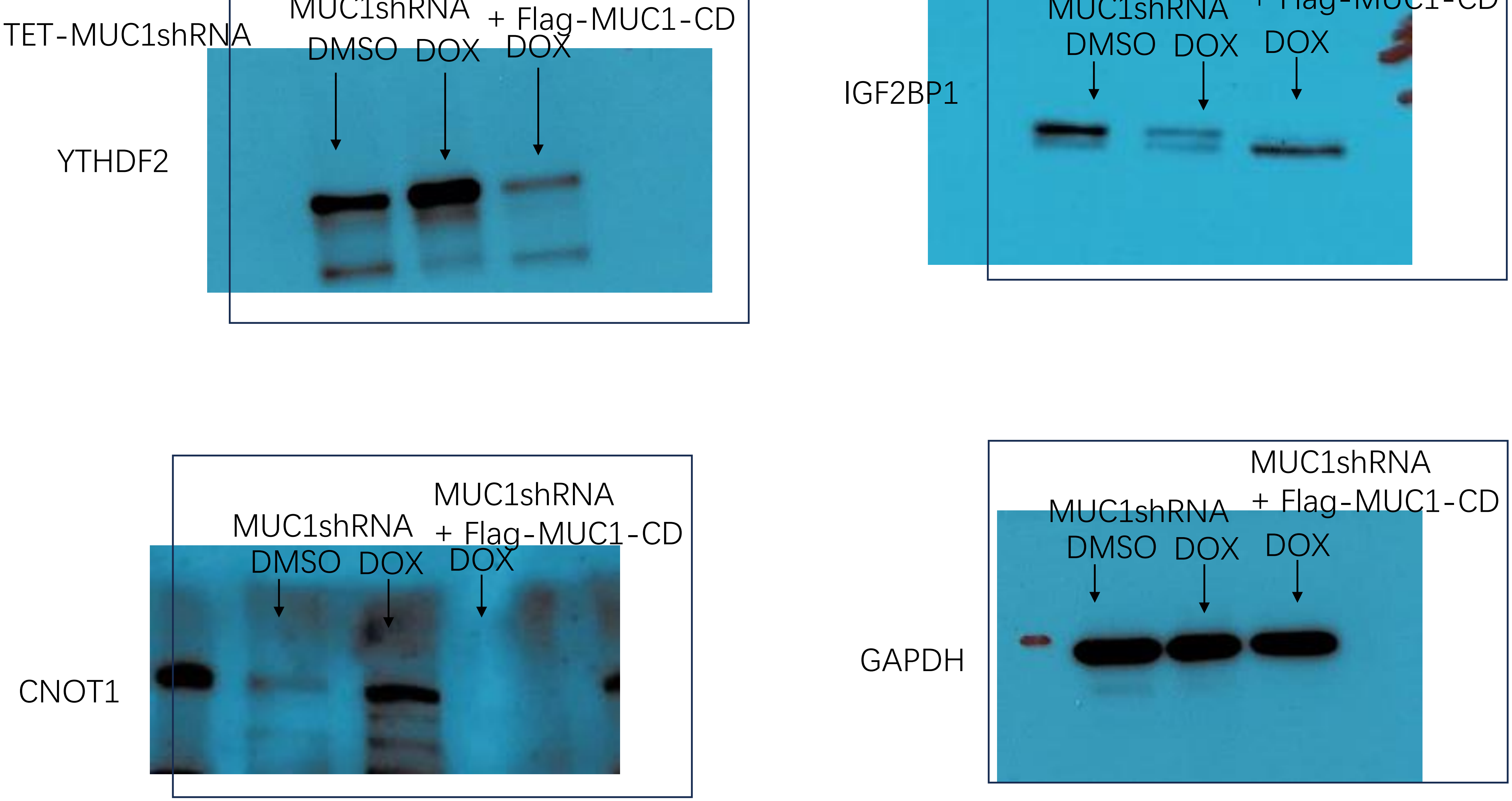
TET-MUC1shRNA
MUC1shRNA
MUC1shRNA
+ Flag-MUC1-CD
DMSO
DOX
DOX
YTHDF2
IGF2BP1
MUC1shRNA
MUC1shRNA
+ Flag-MUC1-CD
DMSO
DOX
DOX
MUC1shRNA
MUC1shRNA
+ Flag-MUC1-CD
DMSO
DOX
DOX
CNOT1
MUC1shRNA
MUC1shRNA
+ Flag-MUC1-CD
DMSO
DOX
DOX
GAPDH

Figure S3 b

TET-MUC1shRNA

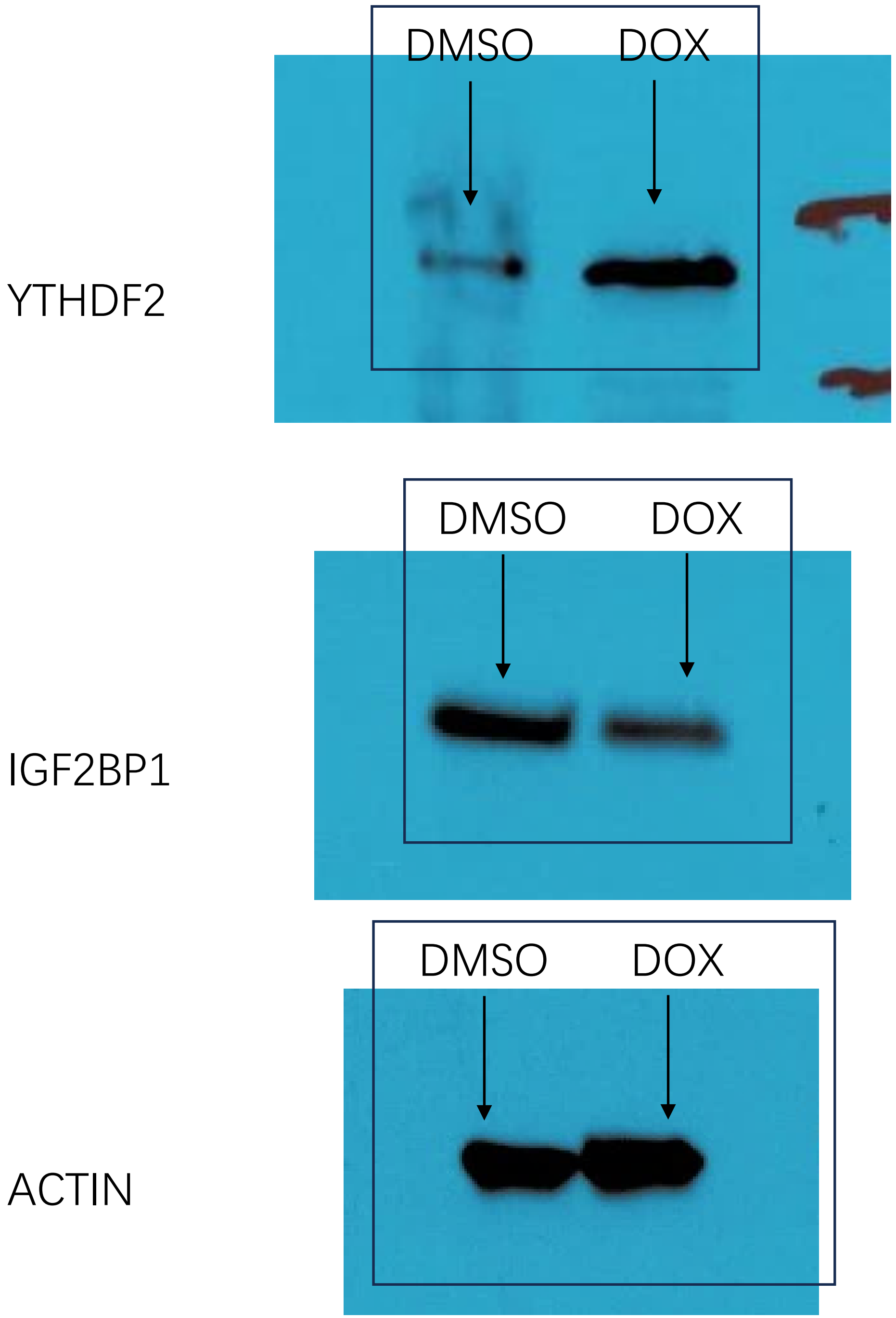

Figure S3 d

TET-MUC1shRNA

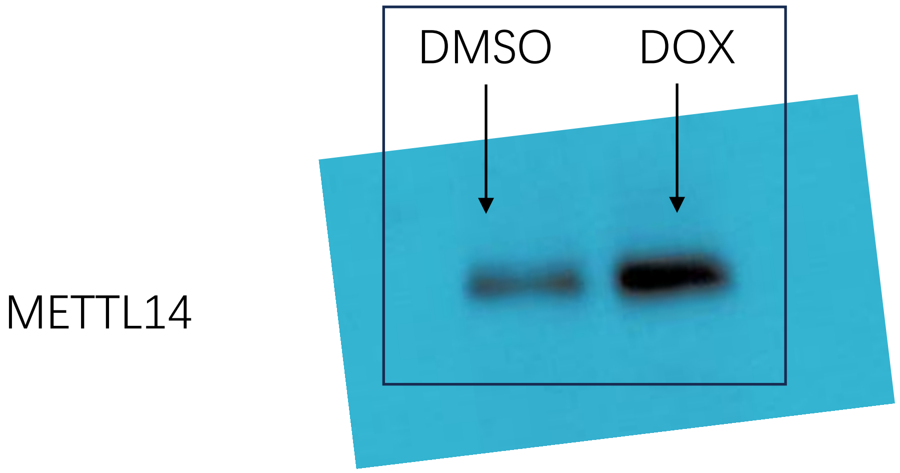

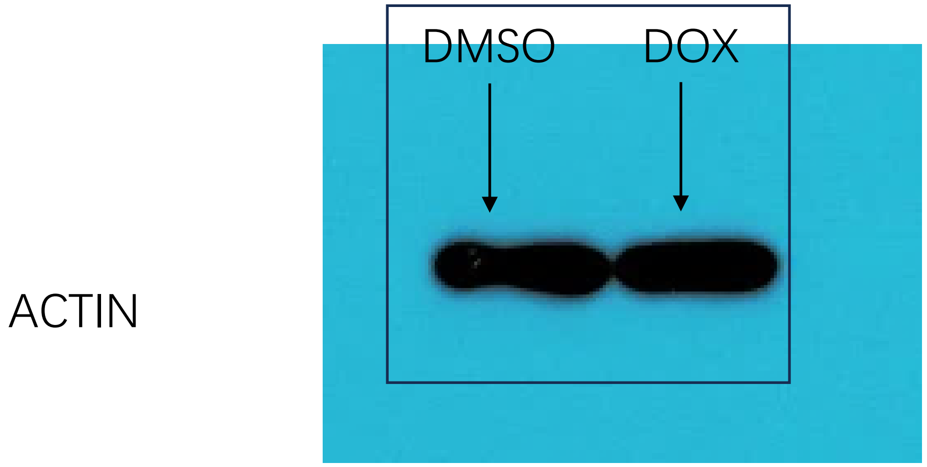

Figure 4 e

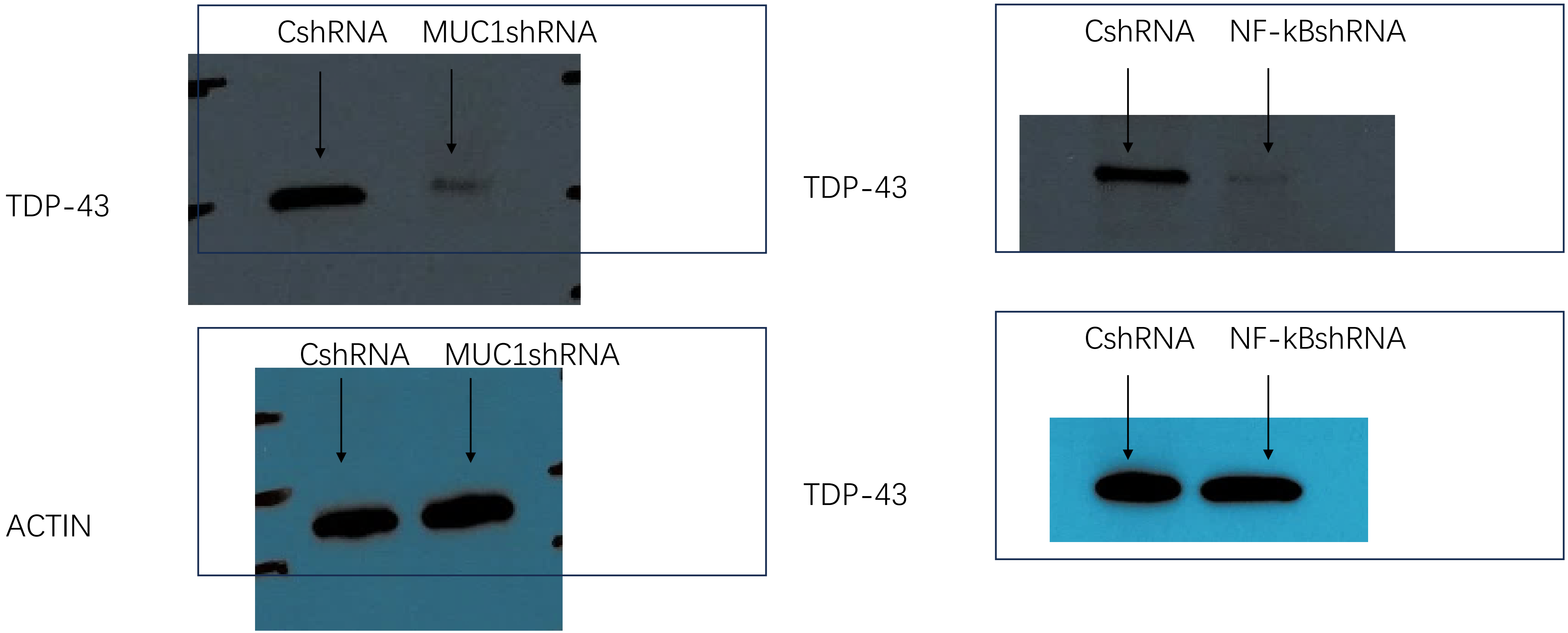
TDP-43
CshRNA
MUC1shRNA
ACTIN
CshRNA
MUC1shRNA
TDP-43
CshRNA
NF-kBshRNA
TDP-43
CshRNA
NF-kBshRNA

Figure 4 f

TET-MUC1shRNA

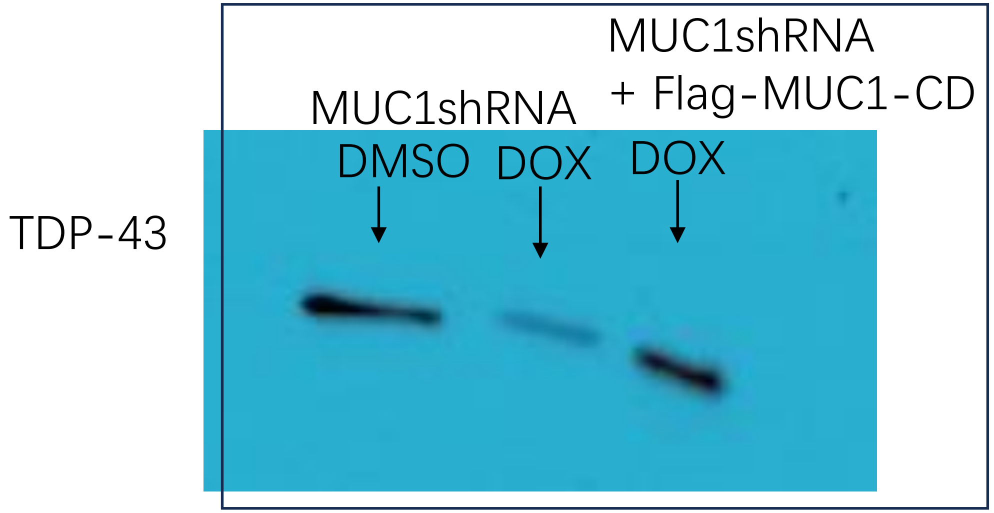

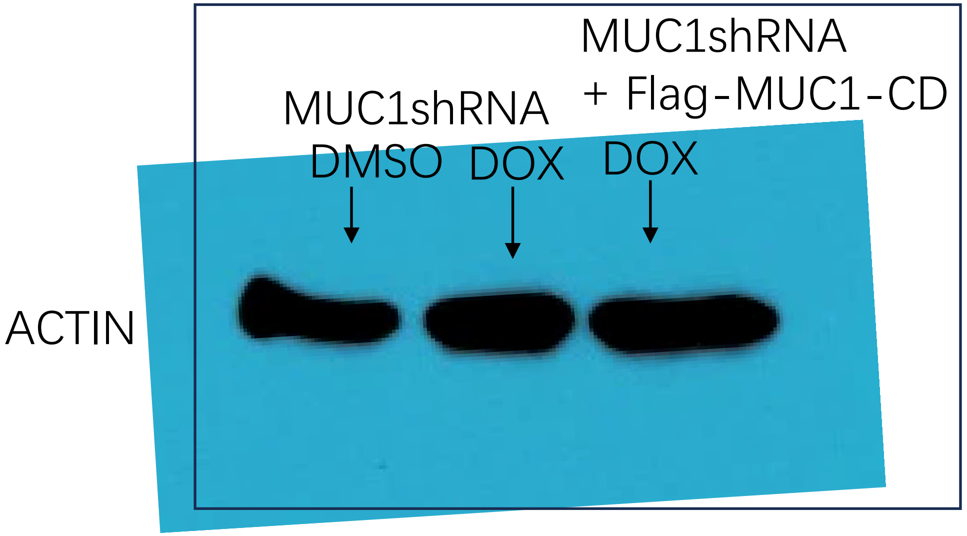

Figure 4 g

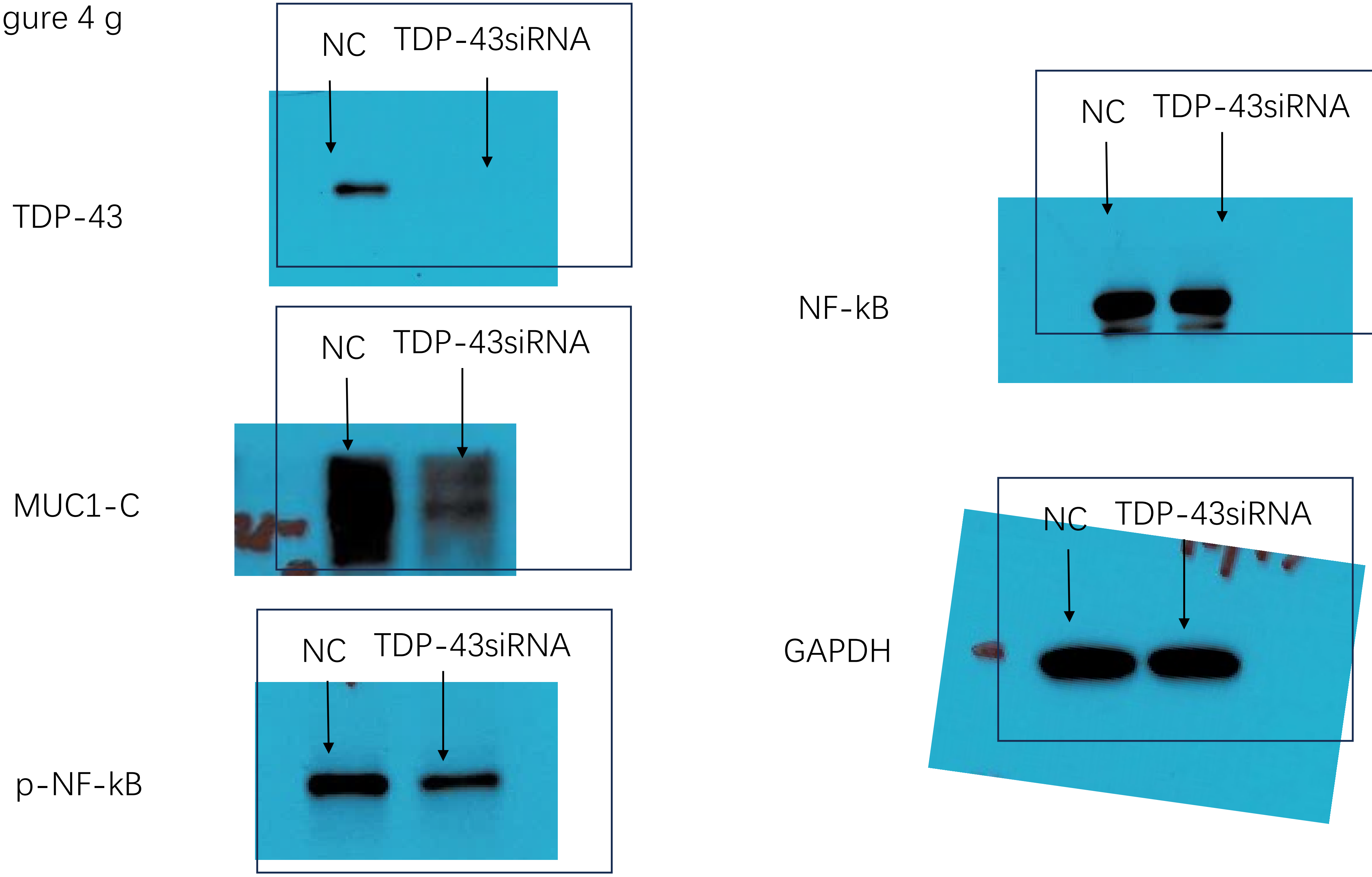

NC
TDP-43siRNA
TDP-43
NC
TDP-43siRNA
MUC1-C
NC
TDP-43siRNA
p-NF-kB
NC
TDP-43siRNA
NF-kB
NC
TDP-43siRNA
GAPDH

Figure S4 c

TET-XISTshRNA

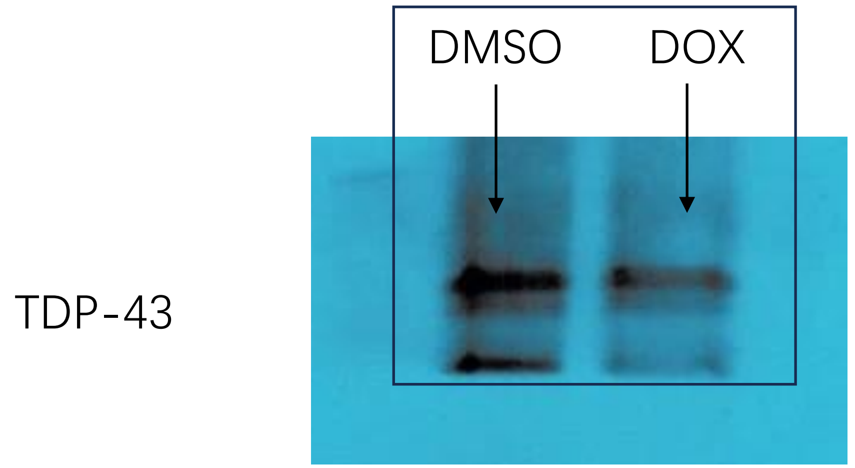

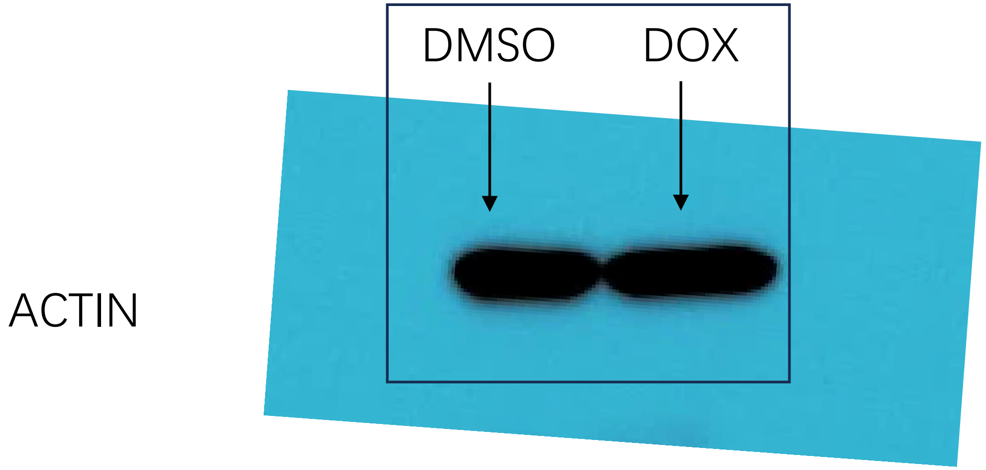

Figure S4 c

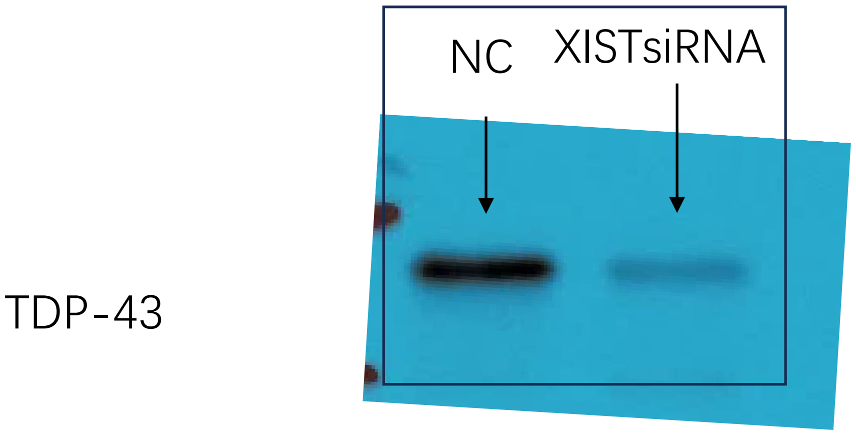

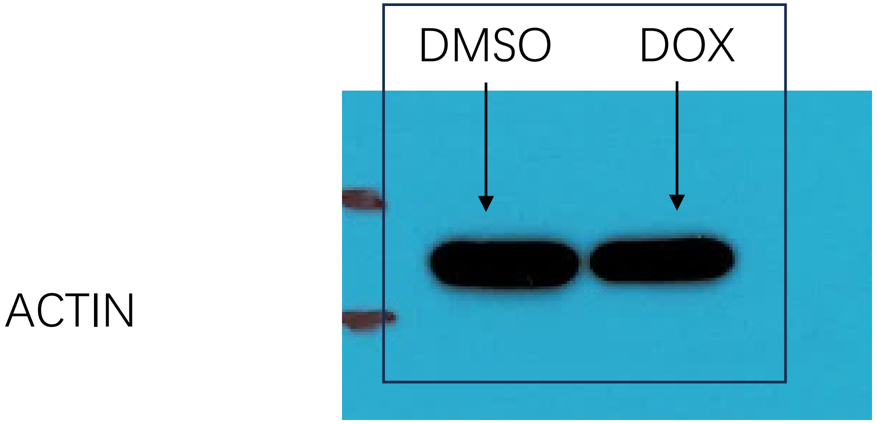

Figure 5 b

TET-XISTshRNA

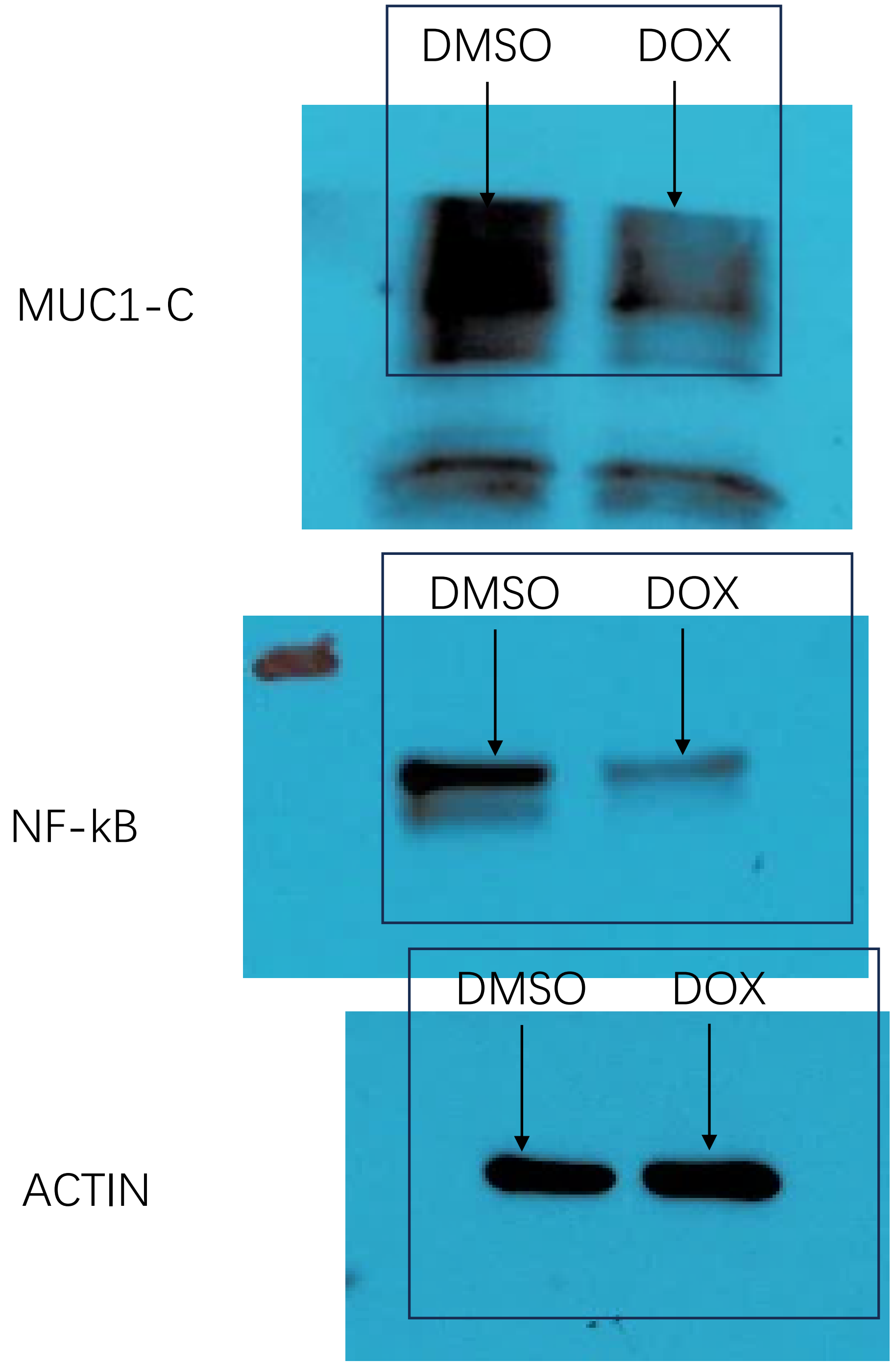

Figure 6 c

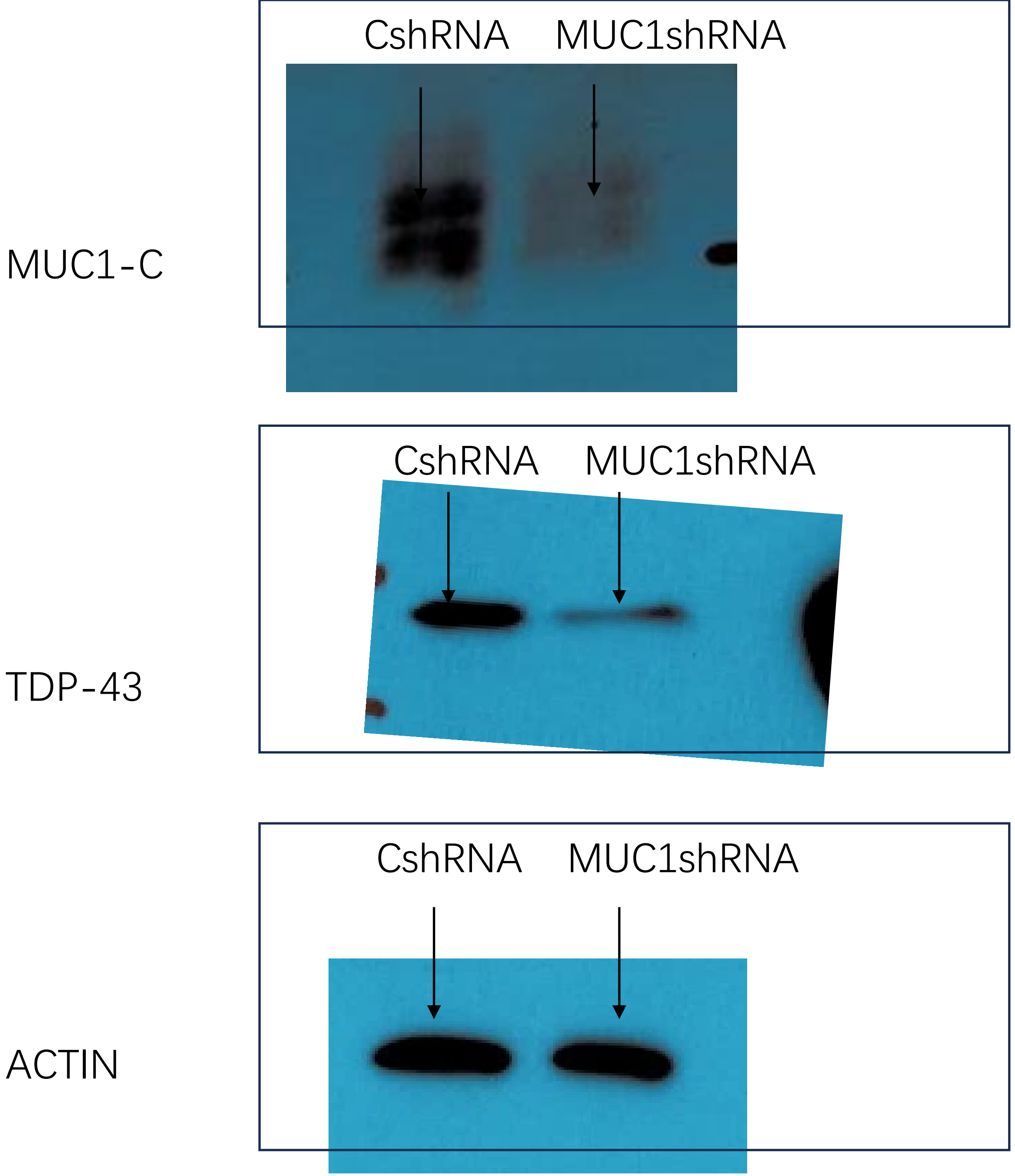
CshRNA
MUC1shRNA
MUC1-C
CshRNA
MUC1shRNA
TDP-43
CshRNA
MUC1shRNA
ACTIN

Figure 6 d

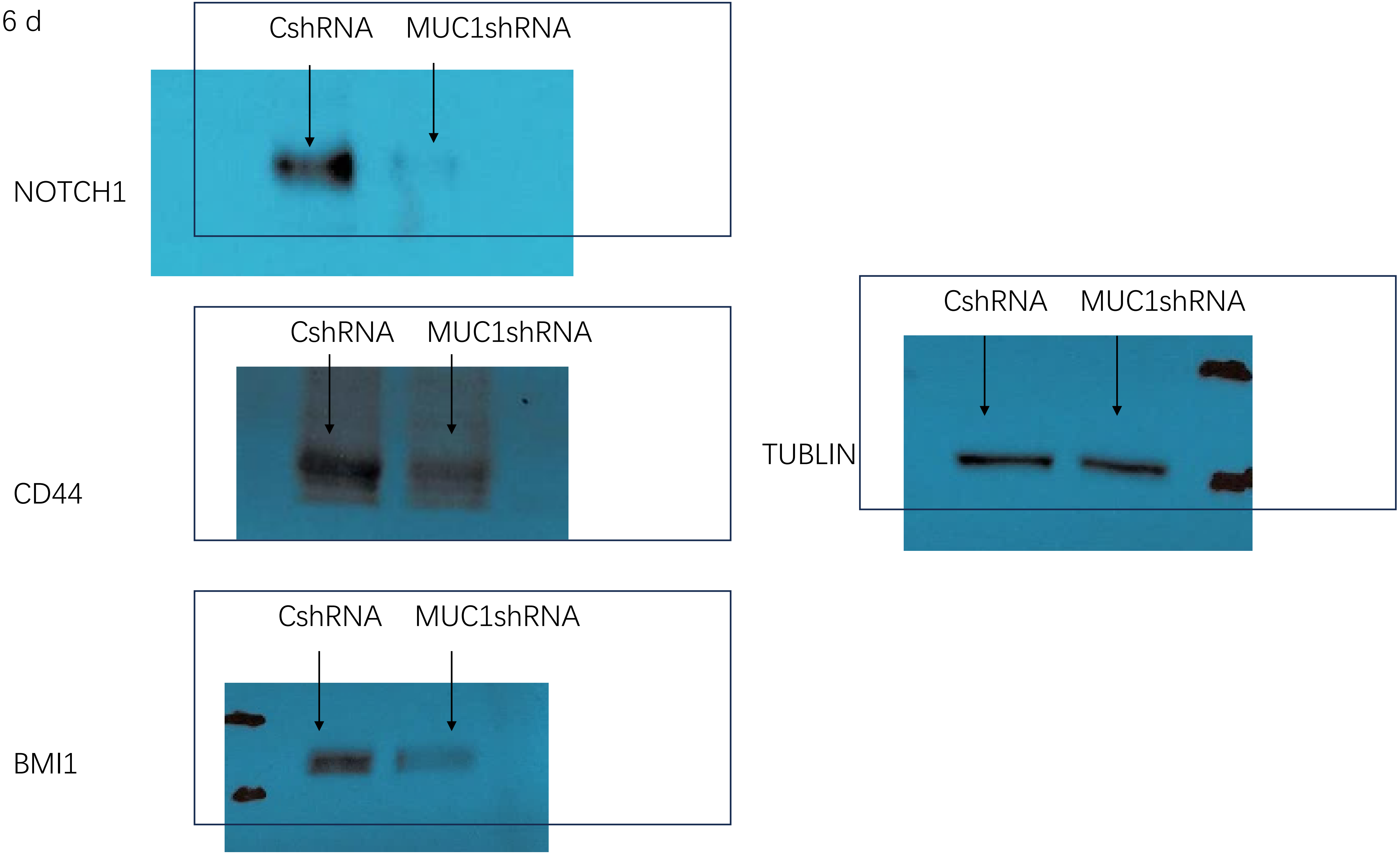
NOTCH1
CshRNA
MUC1shRNA
CD44
CshRNA
MUC1shRNA
BMI1
CshRNA
MUC1shRNA
TUBLIN
CshRNA
MUC1shRNA

Figure 6 e

TET-MUC1shRNA

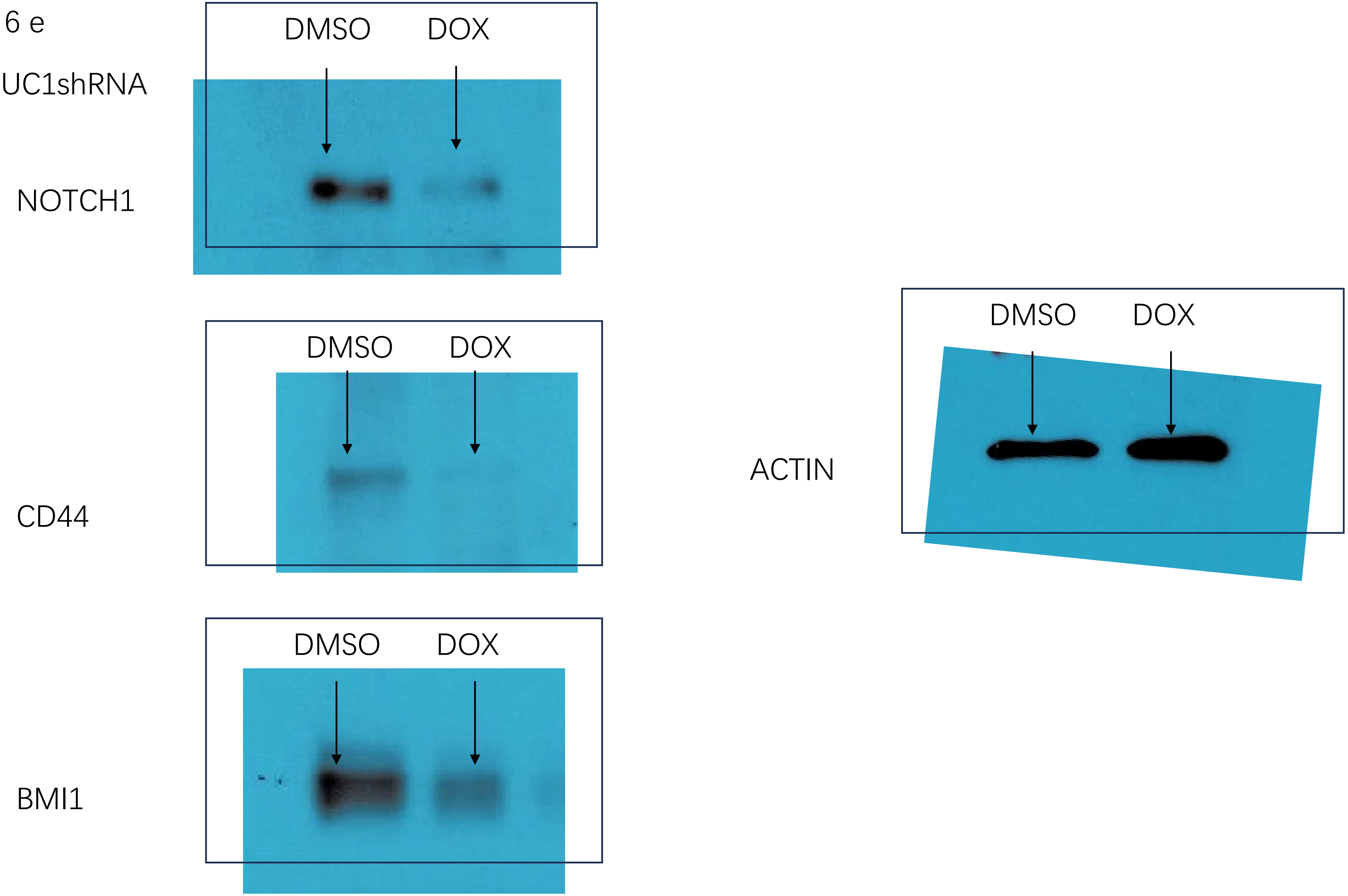

Supplement: Supplementary file 2 — Original Data File [file 41419_2024_6684_MOESM2_ESM.pdf]
